# Simultaneous Amplicon Sequencing to Explore Co-Occurrence Patterns of Bacterial, Archaeal and Eukaryotic Microorganisms in Rumen Microbial Communities

**DOI:** 10.1371/journal.pone.0047879

**Published:** 2013-02-08

**Authors:** Sandra Kittelmann, Henning Seedorf, William A. Walters, Jose C. Clemente, Rob Knight, Jeffrey I. Gordon, Peter H. Janssen

**Affiliations:** 1 AgResearch Ltd, Grasslands Research Centre, Palmerston North, New Zealand; 2 Center for Genome Sciences and Systems Biology, Washington University School of Medicine, St. Louis, Missouri, United States of America; 3 Department of Molecular, Cellular, and Developmental Biology, University of Colorado, Boulder, Colorado, United States of America; 4 Department of Chemistry and Biochemistry and BioFrontiers Institute, University of Colorado, Boulder, Colorado, United States of America; 5 Howard Hughes Medical Institute, Boulder, Colorado, United States of America; University of Illinois, United States of America

## Abstract

Ruminants rely on a complex rumen microbial community to convert dietary plant material to energy-yielding products. Here we developed a method to simultaneously analyze the community's bacterial and archaeal 16S rRNA genes, ciliate 18S rRNA genes and anaerobic fungal internal transcribed spacer 1 genes using 12 DNA samples derived from 11 different rumen samples from three host species (*Ovis aries*, *Bos taurus*, *Cervus elephas*) and multiplex 454 Titanium pyrosequencing. We show that the mixing ratio of the group-specific DNA templates before emulsion PCR is crucial to compensate for differences in amplicon length. This method, in contrast to using a non-specific universal primer pair, avoids sequencing non-targeted DNA, such as plant- or endophyte-derived rRNA genes, and allows increased or decreased levels of community structure resolution for each microbial group as needed. Communities analyzed with different primers always grouped by sample origin rather than by the primers used. However, primer choice had a greater impact on apparent archaeal community structure than on bacterial community structure, and biases for certain methanogen groups were detected. Co-occurrence analysis of microbial taxa from all three domains of life suggested strong within- and between-domain correlations between different groups of microorganisms within the rumen. The approach used to simultaneously characterize bacterial, archaeal and eukaryotic components of a microbiota should be applicable to other communities occupying diverse habitats.

## Introduction

Globally, ruminant-derived methane (CH_4_) contributes 8% of total anthropogenic greenhouse gas emissions and represents an energy loss from feed of up to 10% for the animal [1], [2]. Despite ongoing efforts, no viable CH_4_ mitigation technology exists for pasture-fed ruminants [3]. Ruminant animals rely on a complex intestinal microbiota to convert ingested plant material into energy-yielding products. This symbiosis between the host and rumen microbiota has evolved to make use of grasses with a high content of structural carbohydrates and low nutritional value. Cellulolytic bacteria, anaerobic fungi and some ciliate protozoa are the initial colonisers and decomposers of such fibrous feed [4], [5]. The breakdown of plant polymers is followed by fermentation of the resulting monomers and oligomers. Fermentation end products, such as short-chain fatty acids, are then absorbed by the host across the rumen wall. Methanogenic archaea are beneficiaries of the hydrogen (H_2_) generated during fermentation of feed. Their metabolism allows them to gain energy by reducing carbon dioxide (CO_2_) with electrons from H_2_ oxidation, thereby producing CH_4_. CH_4_ cannot be used by the ruminant and is removed from the rumen by eructation.

To date, deep-sequencing studies of the rumen using phylogenetic marker genes have concentrated on investigating bacterial community composition [6], [7], [8], [9], [10], [11], [12], [13]. Although scientific interest in the structure and function of eukaryotic microbes is increasing [14], rumen studies are largely focused on bacterial and archaeal members. Protozoa and fungi, although far less abundant than the bacteria and archaea in terms of cell numbers, can make up approximately half of the total rumen microbial biomass [15]. Until recently, species identification and physiological characteristics of fungal and ciliate communities colonising the gastrointestinal tracts of herbivores have typically been assessed by using microscopy [16],[17] and cultivation-based techniques [18],[19]. Liggenstoffer *et al*. used metagenomic methods to characterize anaerobic fungal members in the guts of herbivores: they did so by focusing on the internal transcribed spacer (ITS) domain positioned between eukaryotic ribosomal RNA genes, collecting more than 250,000 partial internal transcribed spacer 1 (ITS1) from 33 ruminant and non-ruminant herbivores by multiplex pyrosequencing [20]. Eight novel anaerobic fungal lineages were detected, which accounted for 38.3% of the total number of sequences.

In the present study, we use barcoded pyrosequencing of phylogenetic marker genes to simultaneously characterize diversity in all three domains of life represented in the rumen microbiota: bacteria, archaea, and eukarya (ciliate protozoa and anaerobic fungi). We developed a method that permits adjustment of the template concentration to compensate for species diversity, abundance, and amplicon length of the different microbial groups, and furthermore minimizes nonspecific amplification of DNA originating from ingested plant material and endophytic fungi. Clone libraries constructed in earlier studies enabled us to build phylogenetic frameworks for ciliated protozoa [21] and anaerobic fungi [22] facilitating taxonomic classification of pyrosequencing reads. Our approach maximizes the number of samples that can be analyzed while still achieving satisfactory coverage of diversity, thereby increasing the power of comparisons between experimental treatments and minimizing costs of monitoring microbial communities.

## Materials and Methods

### Rumen sampling

The use of animals, including welfare, husbandry, experimental procedures, and the collection of rumen samples used for this study, was approved by the AgResearch Grasslands, AgResearch Ruakura, and Massey University Animal Ethics Committees, and complied with the institutional Codes of Ethical Conduct for the Use of Animals in Research, Testing and Teaching, as prescribed in the Animal Welfare Act of 1999 and its amendments. Rumen samples were collected as part of a series of feeding trials conducted in New Zealand under permit numbers 06/119 and 06/126 (Massey University, Palmerston North), 11110 modification 775, 12174, and 11975 (all three AgResearch, Grasslands Research Centre, Palmerston North), and 11897 (AgResearch, Ruakura Research Centre, Hamilton). The animals were kept at AgResearch's Grasslands Research Centre and at Massey University, Palmerston North, at Woodlands farm, Invercargill, and at Lye farm, Hamilton.

Eleven rumen samples were collected from sheep (*Ovis aries*), cattle (*Bos taurus*) or deer (*Cervus elephas*) feeding on a variety of different diets by sampling through a fistula, via stomach tubing or after sacrifice under supervision of a veterinarian at AgResearch's Ruakura Research Centre (Table 1). The rumen samples have the following naming convention: Ruminant species abbreviation (C  =  cow, D  =  deer, S  =  sheep), animal identity number, diet abbreviation (GR  =  grain, HY  =  hay, PA  =  pasture, SG  =  summer pasture, SI  =  silage, WG  =  winter pasture), location abbreviation (PN  =  Palmerston North, TK  =  Taranaki, RK  =  Ruakura); for example, sample C5SGPN was obtained from cow 5 feeding on summer pasture in Palmerston North. Additional information on diet, flock, and location of all animals sampled in this study is provided in Table 1 (for more details also see [21],[23],[24]).

**Table 1:** Overview of rumen samples analyzed in this study.

Sample identity	Ruminant	Breed	Diet	Sampling	Herd/Flock	Location
C5SGPN	Dairy cow	Friesian-Jersey	Summer pasture^a^	Fistula	1	PN
C5SIPN	Dairy cow	Friesian-Jersey	Silage^b^	Fistula	1	PN
C6HYPN	Beef cow	Friesian cross	Hay^b^	Fistula	2	PN
C7PATK	Dairy cow	Holstein-Friesian	Pasture^a^	Fistula	3	TK
D3WGPN	Red deer		Winter pasture^a^	Fistula	4	PN
D4SGPN	Red deer		Summer pasture^a^	Fistula	4	PN
S19PAPN	Sheep	Coopworth cross	Lucerne Pasture^b^	Stomach tube	5	PN
S2SIPN	Sheep	Romney	Silage^b^	Fistula	6	PN
S4SGPN^c^	Sheep	Romney	Summer pasture^a^	Fistula	6	PN
S4WGPN	Sheep	Romney	Winter pasture^a^	Fistula	6	PN
S5GRRK	Sheep	Romney	Grain^b^	Sacrifice	7	RK

a animals were grazing freely, ^b^ diets were administered to the animals, ^c^ DNA extracted twice from two subsamples and labelled S4SG1PN and S4SG2PN.

The rumen samples analyzed in this study were obtained from sheep, cattle, and red deer feeding on different diets. Samples were taken via a rumen fistula, via stomach tubing, or at slaughter. Animals with the same herd/flock number were co-housed at the location indicated.

### DNA extraction

DNA was extracted from 30 mg of homogenized, freeze-dried and ground rumen sample after Lueders *et al*. [25]. Briefly, cells were disrupted by combined beat-beating and phenol-chloroform-isoamyl alcohol (25∶24∶1; vol:vol:vol) treatment and subsequent precipitation of proteins with chloroform-isoamyl alcohol (24∶1; vol:vol). DNA was precipitated with 2 volumes of 30% (wt:vol) polyethylene glycol, washed with 70% (vol:vol) ice-cold ethanol and eluted in 100 μl elution buffer (EB; 10 mM Tris, pH 8.5 with HCl). RNA was digested with 50 μg RNase A, and DNA was subsequently cleaned up using the QIAquick PCR purification kit (Qiagen, Hilden, Germany) and eluted in 50 μl EB. Note that duplicate extractions were performed in the case of sheep S4 from Palmerston North on summer pasture in order to estimate the comparability of sub-samples analyzed by 454 Titanium pyrosequencing; these two samples were designated S4SG1PN and S4SG2PN.

### Amplification of target genes and amplicon pooling

Primers used for PCR amplification of bacterial and archaeal 16S rRNA genes, ciliate 18S rRNA genes and fungal ITS1 genes are listed in Table 2. In addition to the group specific primer pairs, we also tested a primer pair that simultaneously amplifies bacterial and archaeal 16S rRNA genes (ArBa; Table 2; [26]). All primers contained the 454 Life Sciences (Branford, CT, USA) adaptors A (5′-CCA TCT CAT CCC TGC GTG TCT CCG ACT CAG-3′) or B (5′-CCT ATC CCC TGT GTG CCT TGG CAG TCT CAG-3′) for Titanium sequencing, and a unique 12-base error-correcting barcode was attached to adaptor A for sample identification [27]. Most primers carried a two-base linker sequence between the barcode and the group-specific primer to avoid differential amplification (Table 2; [28]). Amplification was performed as follows: initial denaturation at 95°C for 2 min; a defined number of cycles (30 for bacteria/archaea/protozoa; 35 for fungi) of denaturation (95°C, 20 s), annealing (for temperatures see Table 2, 20 s) and elongation (65°C, 1 min); and a final 7-min extension at 65°C. For each DNA sample, 76 μl of reaction mix was prepared containing 32 μl of 5 PRIME HotMasterMix (5 PRIME, Gaithersburg, MD, USA) and barcoded and non-barcoded primer in water, each to a final concentration of 0.2 μM. Before addition of template DNA, an aliquot of 19 μl was transferred into a sterile tube to serve as a no-template negative control. The remaining 57 μl of reaction mix were spiked with a total of 120 ng of DNA contained in 3 μl of water, and then divided into 3 aliquots of 20 μl each. After amplification, the triplicate PCR products were pooled, and the correct sizes of PCR products and the absence of signal from negative controls were verified by agarose gel electrophoresis. PCR products of the ArBa primer pair simultaneously targeting bacteria and archaea were purified individually using the Agencourt AMPure XP kit (Beckman Coulter, Brea, CA, USA), quantified using the Quant-iT dsDNA BR assay kit (Invitrogen, Carlsbad, CA, USA) and a fluorometer (BioTek Instruments, Winooski, VT, USA), and subsequently pooled in equimolar concentrations. All other amplicons were purified by gel purification as follows: amplicons of the same target gene and region (i.e., all bacterial, archaeal, ciliate, and fungal amplicons) from the 12 DNA samples were pooled into four separate pools and loaded onto a 1%-agarose gel (wt:vol). Bands were visualized, excised under blue light transillumination, and gel purified with the QIAquick gel extraction kit (Qiagen). Gel-purified amplicon pools were quantified in triplicate with the Quant-iT dsDNA HS assay kit (Invitrogen). To test the feasibility of pooling amplicons from different groups of microorganisms we carried out multiple pyrosequencing runs. The first run contained amplicons obtained with the ArBa primer pair. The second run contained the short amplicons obtained with primer pairs BaS, ArS, and RF (ratio 1∶1∶1) in half A of the picotiter plate, and the long amplicons obtained with primer pairs BaL, ArL, and RP (ratio 1∶1∶1) in half B. Run 3 contained in each half the amplicons of all four groups of microorganisms (bacteria, archaea, protozoa, and fungi) obtained with primer pairs BaL, ArL, RP, and RF, pooled in two different mixing ratios (5∶1∶1∶1 and 5∶1∶1∶0.2). Pools were diluted to obtain a total of 2×10^5^ copies ul^-1^, and emulsion PCR was performed with the Lib-L kit (454 Life Sciences). DNA positive beads were enriched, counted on a Z1 particle counter (Beckman Coulter), and loaded onto a picotiter plate for pyrosequencing on a 454 Life Sciences Genome Sequencer FLX machine (454 Life Sciences).

**Table 2:** Oligonucleotide primers used to amplify bacterial and archaeal 16S rRNA genes, ciliate 18S rRNA genes and anaerobic fungal ITS1 genes.

Name	Adaptor	(Linker^a^ +) Primer sequence [5′ → 3′]	Region	Length [bp]	Annealing [°C]	Abbreviation	Reference
ArBa515F	B	(TA) GTG CCA GCM GCC GCG GTA A	16S	∼290	52	ArBa	[26]
ArBa806R	A^b^	(AC) GGA CTA CVS GGG TAT CTA AT					[57]
Ba9F	B	GAG TTT GAT CMT GGC TCA G	16S	∼525 (+73)	52	BaL	[58]
Ba515Rmod1	A^b^	CCG CGG CKG CTG GCA C					modified from [59]
Ba27F	B	AGA GTT TGA TCC TGG CTC AG	16S	∼365	52	BaS	[60]
Ba338R	A^c^	TGC TGC CTC CCG TAG GAG T					modified from [61]
Ar915aF	A^b^	AGG AAT TGG CGG GGG AGC AC	16S	∼492 (+73)	59	ArL	[62]
Ar1386R	B	GCG GTG TGT GCA AGG AGC					[63]
Ar344F	A^b^	ACG GGG YGC AGC AGG CGC GA	16S	∼148 (+73)	60	ArS	[64]
Ar519R	B	TTA CCG CGG CKG CTG					[65]
RP841F	B	(AA) GAC TAG GGA TTG GAG TGG	18S	∼511 (+73)	54	RP	[21]
Reg1302R	A^b^	(TC) AAT TGC AAA GAT CTA TCC C					[66]
MN100F	A^b^	TCC TAC CCT TTG TGA ATT TG	ITS1	∼250 (+73)	50	RF	[67]
MNGM2	B	CTG CGT TCT TCA TCG TTG CG					[67]

a Linker sequences were designed according to Walters *et al*. [28].

b Golay barcodes of 12-nucleotides in length were used in combination with this primer.

c Hamming barcodes of 8-nucleotides in length were used in combination with this primer.

### Phylogenetic analysis of pyrosequencing reads

Samples were processed and analyzed following the procedure described by Caporaso *et al*. [29] using QIIME v1.2.1. Sequences >200 bp in length were truncated so that the average quality score was >25 and only sequences without ambiguous characters were included in the analysis. An exception was made for the extremely short sequences obtained with the ArS primer pair. Here, filter settings were adjusted to allow for a minimum sequence length of 150 bp. For homopolymer-rich anaerobic fungal ITS1 sequences the option –H 8 was passed to allow for a maximum length of a homopolymer run of eight base pairs (default: –H 6). Sequence reads were assigned to corresponding samples by examining the 8- or 12-nucleotide error-correcting Hamming or Golay barcodes, respectively. OTU picking was performed using uclust [30] for bacteria and archaea at 97% similarity threshold or the prefix-suffix method (QIIME team, unpublished) passing the option “–p 1000” for protozoa and fungi. Sequence data were given phylogenetic assignments as follows: bacterial 16S rRNA genes were blasted against the greengenes database (gg_97_otus_4feb2011.fasta; [31]); archaeal 16S rRNA genes [32], ciliate 18S rRNA genes [21], and fungal ITS1 genes ([22]; version 2.1) were blasted against rumen specific, in-house databases derived from earlier studies (available from the authors upon request). To test whether the databases constructed for anaerobic fungi and ciliate protozoa gave correct taxonomic assignments, two sequence files were created. The only difference between the two sequence files was the order of sequences. In one file, reference sequences were sorted by accession number in alphabetical order, whereas in the other file, the sequences were sorted by accession number in reverse-alphabetical order. Blasting sequences against these two different sequence files resulted in unambiguous taxonomic assignments for almost all sequences. However, a small number of sequences gave ambiguous taxonomic assignments but the differences in abundances caused by these ambiguous assignments were negligible (Table S1). Sequences generated in this study were deposited in the European Bioinformatics Institute (EBI) database under the study accession numbers ERP002012 (Kittelmann_Run_To_Run_Variation), ERP002013 (Kittelmann_Ar_Ba_Primer_Comparison), ERP002014 (Kittelmann_Biology_Bacteria), ERP002015 (Kittelmann_Biology_Archaea), ERP002016 (Kittelmann_Biology_Protozoa), and ERP002017 (Kittelmann_Biology_Fungi).

### Quantitative PCR of bacterial and archaeal 16S rRNA genes in rumen samples

Quantitative PCR (qPCR) of bacterial and archaeal 16S rRNA genes was carried out as described earlier [21],lsqb;23]. Briefly, 3 dilutions (1∶10, 1∶20 and 1∶40) were prepared for each DNA sample, and reactions were set up in duplicate for each dilution.

### Statistical analyses

Differences between bacterial and archaeal communities obtained by using different sets of primers were calculated in QIIME using the Bray-Curtis dissimilarity distance metric, which takes into account presence or absence as well as abundance of OTUs.

Unpaired and paired Student's t-tests using a two-tailed distribution were performed in Excel (Microsoft Corp., Redmond, WA, USA) to evaluate the significance of between-primer/within-sample and within-primer/between-sample dissimilarities of all primer pairs targeting bacteria and archaea, and to evaluate bias for or against certain groups of archaea by the three primer pairs tested (ArL, ArS, and ArBa), respectively.

Comparison of results from pyrosequencing libraries constructed with the ArBa primer pair with those of qPCR of bacterial and archaeal 16S rRNA genes in the same samples was carried out using the CORREL and FDIST functions in Excel, and statistics of fit were calculated according to Goodman [33].

Statistical analyses including rarefaction curves, beta-diversity estimates (Bray-Curtis, Sørensen-Dice) and UPGMA cluster analyses were conducted using the QIIME pipeline [29]. Beta-diversity was evaluated using family level taxa for bacteria [31] and at an operational clade level for archaea [32]. For the comparison of primer pairs, groups showing an abundance of less than 1% of the total community were excluded from the analysis. Rarefaction analysis was performed using rarefied OTU tables (rarefied to the lowest number of reads obtained for any of the 12 DNA samples analyzed), 100 replications, and cut-offs of 97% and 95% sequence similarity, respectively [34].

Simpson's index of dominance (1-D) describes the diversity of a community with 1 ( = 100%) indicating maximum diversity in a sample. This index was calculated according to Simpson [35] using the software PAST [36].

Co-occurrence analysis between microbial populations was performed in the R studio v0.94.110 [37]. Only microbial groups that represented ≥1% of the total community within each of the four microbial groups (bacteria, archaea, protozoa or fungi) in at least one sample and that were detected in ≥50% of rumen samples were included in the analysis. Spearman's rank correlations and *P*-values were calculated and plotted using the packages ellipse [38], hmisc [39], and corrplot [40].

## Results and Discussion

### Diversity of microbial groups and amplicon mixing

To study the composition of bacterial, archaeal and eukaryotic components of the rumen microbiota in New Zealand ruminants, we used the data collected with the primer sets giving the longest amplicons, as these allow for the best possible phylogenetic resolution. Rarefaction analysis was carried out at arbitrary OTU cut-offs of 95% and 97% similarity to estimate the average number of sequences of each microbial group that had to be sampled to describe phylogenetic diversity across all samples [34]. Rarefaction curves of the archaeal, ciliate and anaerobic fungal communities started to level off markedly after approximately 1,000 sequences had been sampled (Figure S1). Therefore, we concluded that archaeal, ciliate and anaerobic fungal diversity in rumen samples would be adequately compared by generating 1,000 sequences per sample. In contrast, rarefaction curves of bacteria never leveled, even after 5,000 sequences had been sampled (Figure S1). However, our data show that approximately 5,000 sequences are sufficient to distinguish between bacterial communities in different samples (Figures S2A, S2B and S3A).

**Figure 1:**
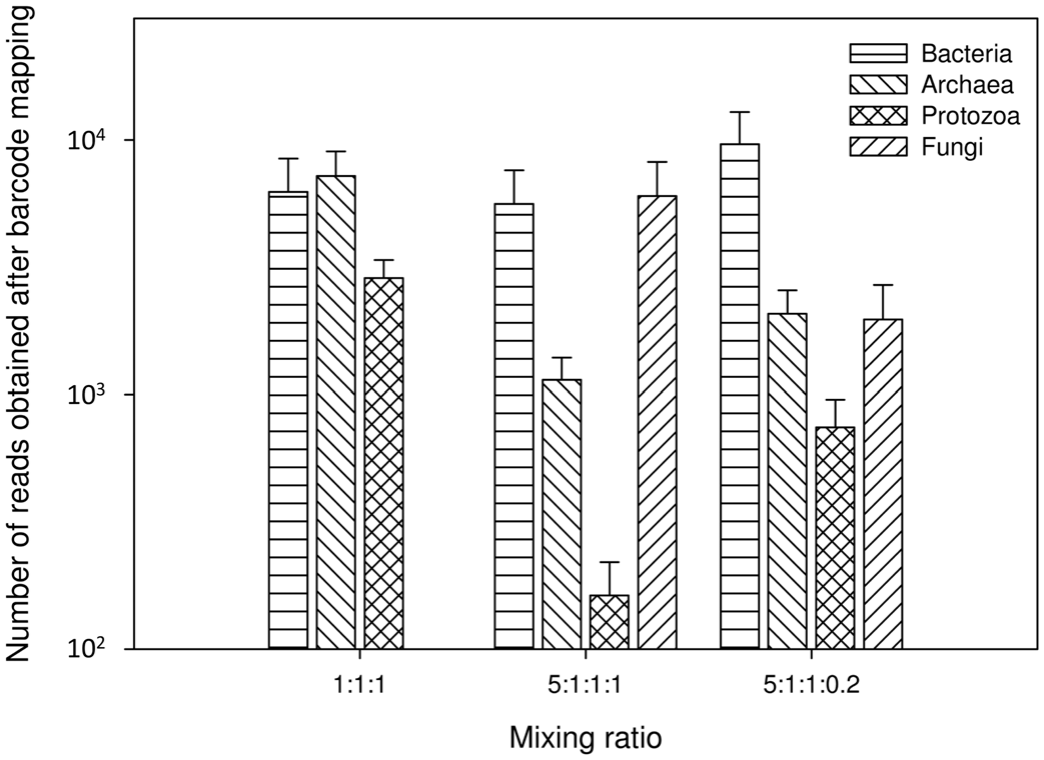
Distribution of barcode mappable and quality-filtered sequencing reads across domains for each mixing ratio tested. The mixing ratios represent bacteria:archaea:ciliate protozoa (1∶1∶1), and bacteria:archaea:ciliate protozoa:fungi (5∶1∶1∶1 and 5∶1∶1∶0.2).

Amplicons were mixed at different ratios to compensate for the different degrees of diversity within the four microbial groups and for the differing amplicon lengths. Mixing only the long amplicons in a ratio of 1∶1∶1 resulted in a similar number of assignable reads for bacteria (74,938; 598 bp amplicon) and archaea (86,502; 565 bp amplicon), but only 34,369 reads for ciliate protozoa (584 bp amplicon; Figure 1). We then added the fungal ITS1 amplicons to analyse all four groups simultaneously. To allow a more extensive sampling of their higher phylogenetic diversity, we added 5 times the amount of bacterial template DNA as compared to archaea, ciliate protozoa and anaerobic fungi (5∶1∶1∶1). From this, we obtained 67,264 reads for bacteria, 13,670 reads for archaea, 1,949 reads for ciliate protozoa and 72,137 reads for anaerobic fungi (210–290 bp amplicon; Figure 1). Although the fungal templates were 5 times underrepresented as compared to the bacterial templates, the sequence yield was similar for these two groups. This result is likely to be due to a preferential amplification of smaller products during emulsion PCR [41]. Fungal diversity in the rumen, however, is much lower than that of the Bacteria and comparable to that of the archaea and ciliate protozoa [22]. Because we did not need the high level of coverage of fungal sequences, we therefore performed a third mixing ratio test, in which we maintained the ratio of bacteria, archaea and ciliate protozoa but reduced the amount of fungal templates (5∶1∶1∶0.2). This ratio resulted in 115,283 reads for bacteria, 24,881 reads for archaea, 8,917 reads for ciliate protozoa, and 23,637 reads for anaerobic fungi (Figure 1). Figure S4 shows the distribution of pyrosequencing reads across the different amplicon lengths for both mixing ratios tested.

An amplicon mixing ratio of 5∶1∶1∶0.2 achieved an approximate average of 5,000 sequence reads for bacteria, and 1,000 sequence reads for archaea, ciliate protozoa and anaerobic fungi for the 12 rumen DNA samples analyzed in this study. This ratio would allow up to 100 rumen samples to be processed for a single 454 pyrosequencer run (≥400 amplicons; 4 microbial groups ×100 samples); assuming a total yield of 1,000,000 reads per run).

Simultaneous analysis of microbial communities from all three domains of life has been performed before [42]. However, the authors used a single universal primer pair [43] and recovered 71.9% of bacterial, 7.8% of archaeal, and 5.5% of eukaryotic sequences. 14.8% of sequences obtained were of chloroplast or mitochondrial origin. In the rumen environment, simultaneous whole community pyrosequencing using amplicon mixing holds several advantages compared to using a single universal primer pair. *First*, this approach allows for the selection of different target genes that provide sufficient phylogenetic resolution for all groups studied, rather than relying on one conserved gene for all groups of interest. *Second*, it provides specificity against amplification of unwanted co-extracted DNA, such as plant- or endophyte-derived DNA. *Third*, amplicon mixing allows compensation for three important variables: (i) the abundance of each microbial group in the environment studied; (ii) the degree of diversity of each of the groups analyzed; and (iii) the amplicon lengths of selected marker genes. However, simultaneous analysis of amplicons of very different lengths requires careful control of all steps in the process, and it is easy to get massive over- or under-representation of shorter amplicons.

The between-run variation, measured as the dissimilarity between two identical DNA samples in two different runs (mean dissimilarity 5.5%±0.7% (standard deviation)), was smaller than when the same rumen sample was extracted in duplicate and amplicons analyzed in the same 454 run (Text S1; compare S4SG1PN and S4SG2PN in Figure S5; 6.3%±1.2%).

Libraries generated using three different primer pairs that amplified 16S rRNA genes from members of the domain Bacteria clustered by sample rather than by primer pair, indicating that all primer pairs resulted in similar bacterial community compositions (Text S2, Figures S2A, S2B, S3A, Tables S2, S3). In contrast, when samples were amplified with different primer pairs targeting the domain Archaea, the three libraries generated from each of the samples clustered considerably further apart from each other than did bacterial communities from the same samples (Text S2, Figures S2C, S2D, S3B, S6, Tables S2, S4).

The overall trend of bacterial, and thus archaeal abundances in the 12 pyrosequencing libraries constructed using the ArBa primer pair was correlated with the results obtained from qPCR (Pearson correlation coefficient  = 0.83, *P*<0.001; Text S3, Figure S7), indicating that generalized between-sample comparisons can be validly made from data generated using the ArBa primer pair.

### Composition of bacterial, archaeal and eukaryotic communities in the rumen

To analyse the phylogenetic composition of microbial communities in the 12 rumen DNA samples, data obtained from three sequencing reactions (sequencing runs 2 and 3) per primer set were combined into a single file for each microbial group. Data for Bacteria (BaL), Archaea (ArL) and ciliate protozoa (RP) resulted from sequencing runs 2 (half B) and 3 (both halves). Data for anaerobic fungi (RF) resulted from sequencing runs 2 (half A) and 3 (both halves). All four data files were analyzed individually using the QIIME software package [29]. In total, we analyzed 257,485 reads for Bacteria (a mean of 21,457 reads per sample; range 16,554–44,221 reads), 125,052 reads for Archaea (mean 10,421; range 7,827–15,998), 45,231 reads for ciliate protozoa (mean 3,769; range 2,785–5,374), and 186,485 reads for anaerobic fungi (mean 15,540; range 7,053–24,438).

**Figure 2:**
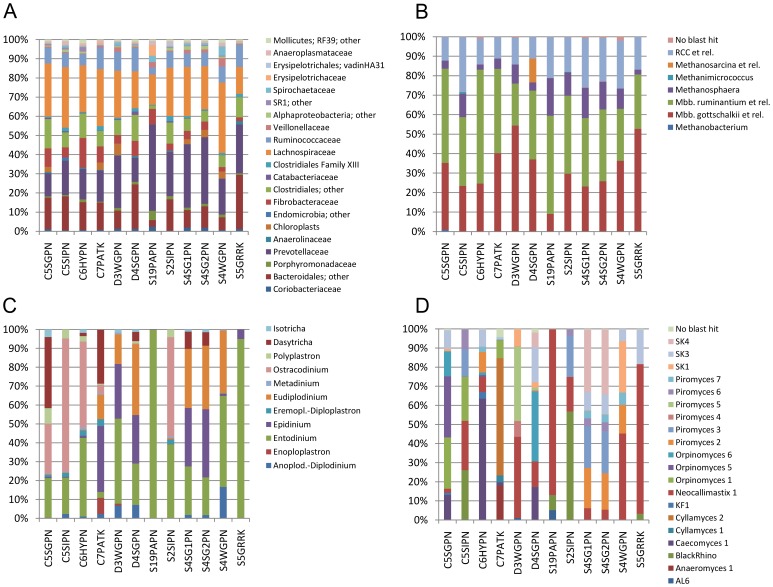
Relative composition of microbial communities in the 12 pyrosequencing libraries. Community composition of (A) bacteria, (B) archaea, (C) ciliate protozoa, and (D) anaerobic fungi in 12 DNA samples obtained from three different ruminant species feeding on a variety of natural diets.

Bacterial communities in the rumen samples were analyzed at the family level and all had a similar diversity (range of Simpson's index of dominance (1-D)  = 75.4–85.2%). Bacterial community composition was very similar across all samples analyzed (mean similarity between samples using Sørensen-Dice 66.7%, range 53.5%–81.2%; mean similarity using Bray-Curtis 73.2%, range 49.7–94.6%; Figure 2A). The two replicates of DNA samples S4SG1PN and S4SG2PN, extracted from the same rumen sample S4SGPN, shared the highest similarity of all between-sample comparisons (81.2% using Sørensen-Dice; 94.6% using Bray-Curtis). The largest groups of bacteria in these 11 rumen samples were classified into the family *Lachnospiraceae*, which made up 13.6 to 40.0% of all bacterial 16S rRNA gene sequences amplified (mean  = 24.0%), and the family *Prevotellaceae*, at 11.6 to 44.9% of all sequences (mean  = 22.9%). These bacteria apparently play a major role in feed degradation in the rumen. The other abundant groups of bacteria were members of the families *Ruminococcaceae* (3.7 to 11.6%; mean  = 8.1%) and *Fibrobacteraceae* (1.9 to 15.1%; mean  = 6.1%), and less well classified groups of the orders *Clostridiales* (1.8 to 14.8%; mean  = 8.6%) and *Bacteroidales* (3.5 to 27.5%; mean  = 13.7%). These six largest groupings accounted for 77.3 to 89.2% of all sequences in all samples (mean  = 83.6%), and were detected in all 11 rumen samples. However, in all cases, their relative abundances varied, by up to 8-fold over the samples, showing that there were some differences in bacterial community structure between the different ruminants, herds or flocks, and diets.

**Figure 3:**
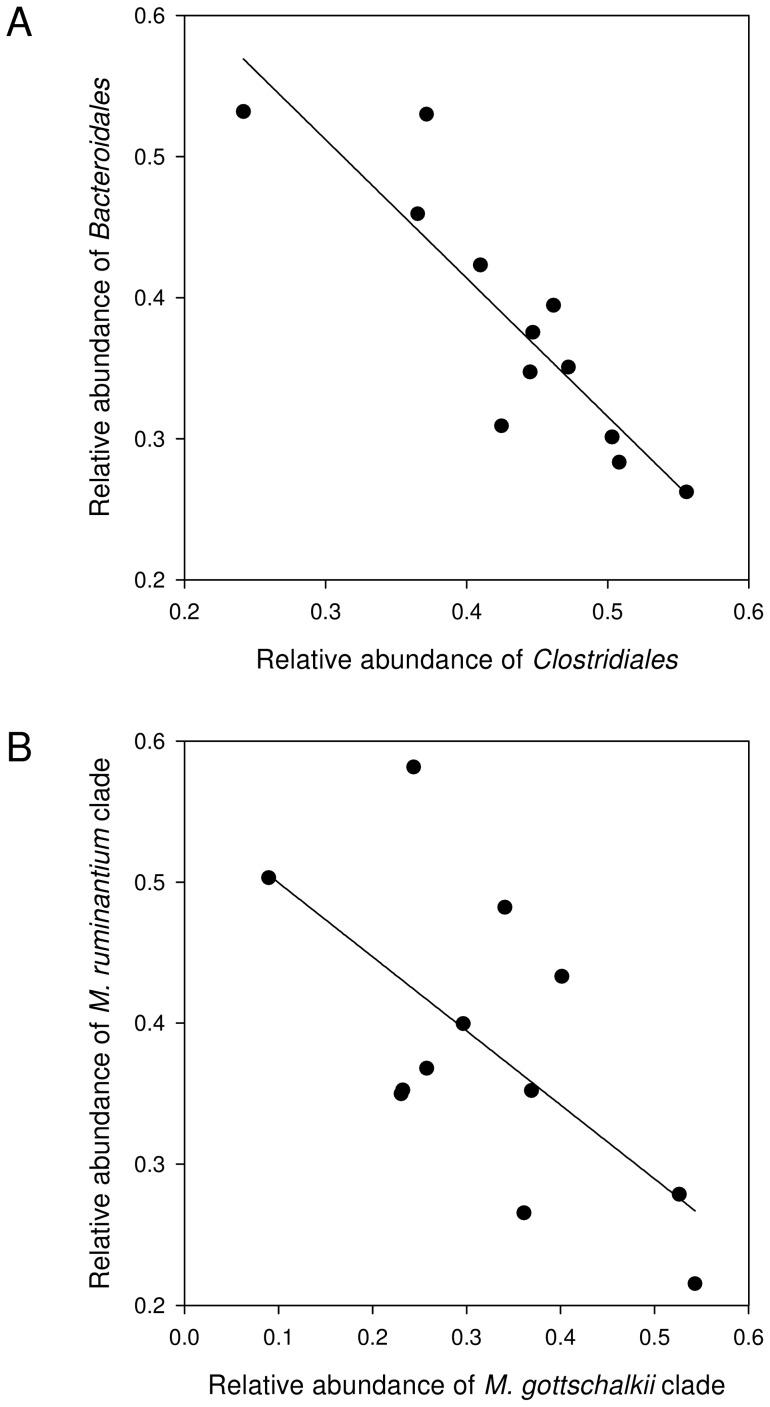
Correlations between selected groups of microorganisms. Correlation between relative abundances of (A) *Bacteroidales*- and *Clostridiales*-related sequencing reads, and (B) methanogens of the *Methanobrevibacter ruminantium* and *M. gottschalkii* clades in pyrosequencing libraries of the 12 analyzed DNA samples.

There was an apparent systematic tradeoff between the abundance of members of the order *Clostridiales* (families *Lachnospiraceae*, *Ruminococcaceae*, *Veillonellaceae*, and *Clostridiales* Family XIII plus less well defined members of the order *Clostridiales*) on the one hand and members of the order *Bacteroidales* (families *Prevotellaceae* and *Porphyromonadaceae* plus less well defined *Bacteroidales*; Figure 3A). This limited diversity of rumen bacteria mirrors that found by others. Using 16S rRNA gene amplicon pyrosequencing, Pitta *et al*. detected bacterial species belonging to a total of only 13 different family-level taxa in cows feeding on Bermudagrass hay or wheat [8]. Similarly, bacterial communities in rumen fluid of cattle feeding on a high grain diet were composed of species belonging to only 13 different families [7]. In agreement with our study, microorganisms belonging to the *Clostridiales* and *Bacteroidales* were also the predominant bacterial groups in steers and cows feeding on grass-legume hay or on switchgrass [6],[11].

In the present study, minor groups detected included *Cyanobacteria* (2.2%±2.1%, mean ± standard deviation), *Proteobacteria* (1.5%±0.9%), *Actinobacteria* (1.4%±0.7%), and *Spirochaetes* (1.3%±1.4%). Chloroplasts contributed 91.1%±5.2% to total cyanobacterial sequence types (in samples where *Cyanobacteria* made up ≥1% of the total bacterial community). No chloroplasts were detected in samples of animals fed a grain- or silage-based diet, in which fewer chloroplasts were expected anyway because of the nature or the treatment of the feed.

The diversity of methanogenic archaea was measured at “operational clade” level, and 1-D ranged from 58.0–73.5%. As with Bacteria, archaeal community composition was similar across all samples analyzed (mean similarity between samples using Sørensen-Dice 79.2%, range 62.5%-100%; mean similarity using Bray-Curtis 77.9%, range 54.2–95.5%). The replicate DNA samples S4SG1PN and S4SG2PN from the same rumen sample shared the highest similarity of all between-sample comparisons (100% using Sørensen-Dice; 95.5% using Bray-Curtis). Sequences clustering within the *Methanobrevibacter ruminantium* clade (38.2%±10.6%), *M. gottschalkii* clade (32.4%±12.9%), Rumen Cluster C (18.1%±6.0%), and the genus *Methanosphaera* (9.3%±5.5%) were most abundant (Figure 2B). Besides these groups, sample D4SGPN from a red deer consisted of a large number of sequences derived from *Methanosarcina* species (12.2%). Out of nine *Methanosarcina*-related OTUs that had an abundance of ≥10 reads in this sample (making up 958 from a total of 1,005 *Methanosarcina*-related reads), 50.7% and 32.5% were ≥99% similar to homologous regions of 16S rRNA genes from *M. barkeri* and *M. mazei*, respectively. This finding agrees with the detection of a significant population of *Methanosarcina* spp. in the same animal, a deer on pasture in summer using different methods [23]. *Methanosarcina* spp. are usually rare in the rumen [32], because of their low growth rates, despite the abundance of acetate which is a growth substrate for these methanogens. *Methanosarcina* spp. are known to occur in large numbers when rumen liquid turnover is low [44]. Interestingly, deer are reported to slow down the passage rate of particles through the rumen in summer but not in winter, compared to other ruminants [45].

Of the 11 rumen samples analyzed in this study, 8 were also analyzed using denaturing gradient gel electrophoretic separation of partial 16S rRNA amplicons and by clone libraries using different primers by Jeyanathan *et al*. [23]. The pyrosequencing approach showed that the same methanogen groups were present in these samples. The increased sample size using pyrosequencing indicated the presence of other groups not detected by Jeyanathan *et al*. [23], but these made up only 0.63% of all pyrosequencing reads in those libraries.

Ciliate communities in the samples analyzed showed a broad range of diversity at the genus level (range 1-D  = 0.4–76.7%), and ciliate community structure was very different between the different samples analyzed (41.4% average between-sample similarity using Bray-Curtis; Figure 2C). Interestingly, the highest similarity between samples was not obtained for the two replicates of sample S4SGPN (92.3%), but for samples S19PAPN and S5GRRK (95.1%). This result can be explained by the low diversity of samples S19PAPN (1-D  = 0.4%) and S5GRRK (9.6%) as compared to the much higher diversity in samples S4SG1PN (73.1%) and S4SG2PN (71.1%). Species of the genus *Entodinium* were the most abundant group of ciliates, with an average abundance of 40%, confirming earlier reports based on microscopic counting [46]. Four of the 11 rumen samples analyzed showed A-type ciliate communities [47], which are characterized by the presence of *Polyplastron multivesiculatum* (C5SGPN, C5SIPN, C6HYPN, and S2SIPN). These communities were dominated by *Ostracodinium*, *Dasytricha* and *Entodinium* species. B-type ciliate communities, characterized by the presence and dominance of *Epidinium* and *Eudiplodinium* spp. [47], were observed in six rumen samples (C7PATK, D3WGPN, D4SGPN, S4SGPN, and S4WGPN). The two remaining rumen samples harboured O-type ciliate communities [47], consisting almost entirely of *Entodinium* species (≥94.4%; S19PAPN and S5GRRK). These findings confirm that the ciliate community types identified by Eadie [47] using microscopy can be easily detected and distinguished using this molecular ecological approach.

The diversity of anaerobic fungal communities in the analyzed rumen samples was compared at cluster level as defined by Kittelmann *et al*. [22]. Anaerobic fungal diversity based on Simpson's index of dominance ranged from 23.9–78.5%. The low average between-sample similarity (21.6%) indicated the much higher variability of anaerobic fungal communities as compared to the bacteria (73.2%), archaea (77.9%) and even ciliate protozoa (41.4%; Figure 2D). The replicate DNA samples S4SG1PN and S4SG2PN showed highest between-sample similarity using the Bray-Curtis metric (97.1%). Major phylogenetic groups belonged to the genera *Neocallimastix* (28%), *Piromyces* (20%), the novel clades SK1, SK3, and SK4 (16%; [22]), *Orpinomyces* (12%), BlackRhino (8%), *Caecomyces* (8%) and *Cyllamyces* (5%).

### Co-occurrence of microbial populations in the rumen

Microorganisms from all three domains of life form a complex network in the rumen ecosystem to ferment the fibrous plant material ingested by the ruminant. The different microbial groups in the rumen have been studied separately for various aspects such as the influences of diet, ruminant host species and other factors on the microbial populations present. To the best of our knowledge, only a single study exists where community composition of bacteria, archaea, ciliate protozoa and anaerobic fungi in the rumen was studied as a whole [48], but the use of different analysis techniques for each microbial group hindered the detection of potential cross-domain interactions.

**Figure 4:**
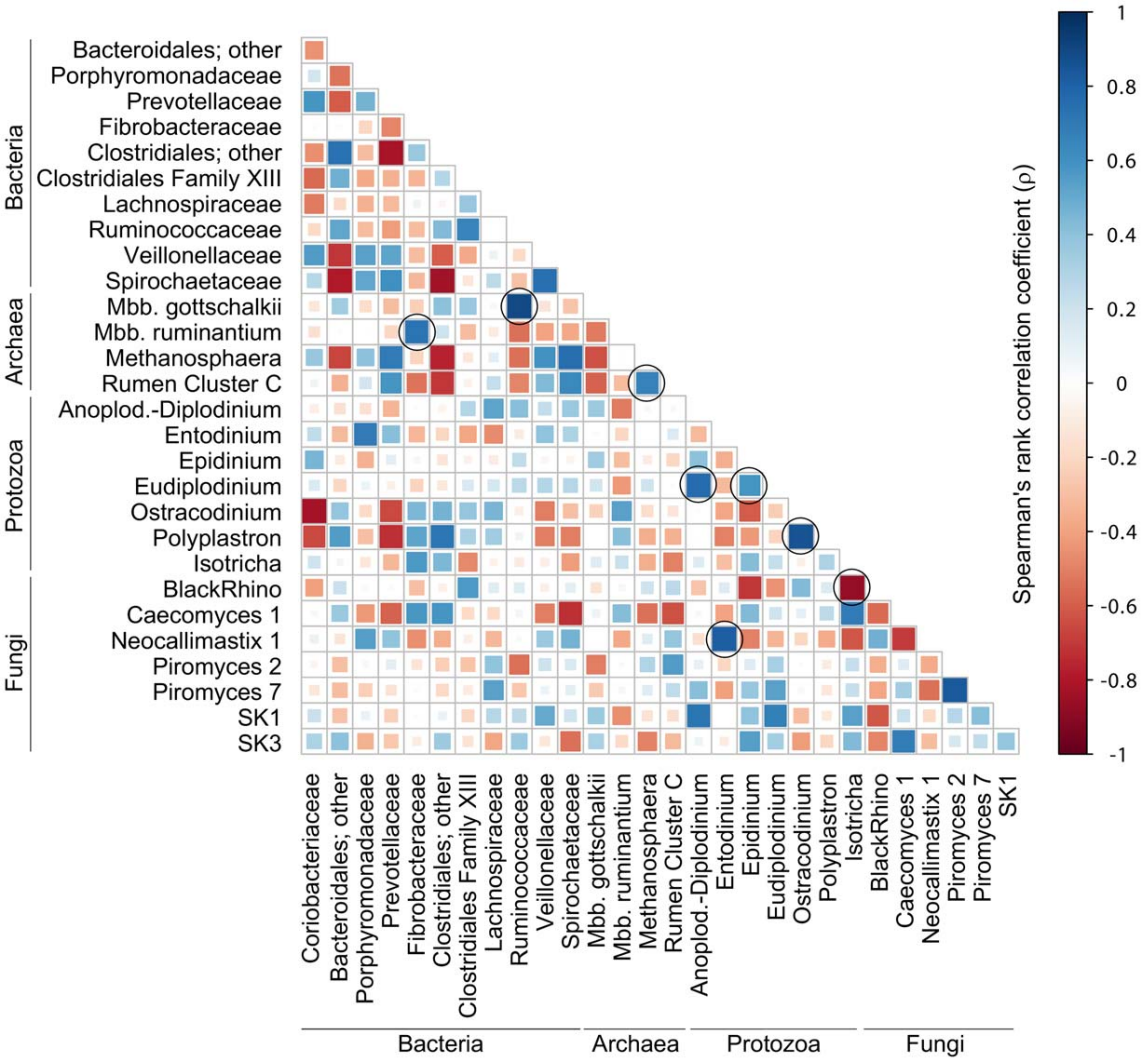
Spearman's rank correlation matrix of the dominant microbial populations across domains in analyzed rumen samples. Microbial populations listed represent at least 1% of the bacterial, archaeal, ciliate, or fungal communities in at least one sample and were detected in at least 50% of the rumen samples analyzed. Strong correlations are indicated by large squares, whereas weak correlations are indicated by small squares. The colours of the scale bar denote the nature of the correlation with 1 indicating perfect positive correlation (dark blue) and -1 indicating perfect negative correlation (dark red) between two microbial populations. Correlations marked with circles are discussed in the text.

Here, we describe microbial co-occurrence patterns in the analyzed rumen samples using the combined data sets (as described in the previous section). Across all samples, our analysis showed a general negative correlation between methanogens of the *Methanobrevibacter ruminantium* clade and those of the *Methanobrevibacter gottschalkii* clade (R = −0.51 [Spearman's rank correlation coefficient], *P* = 0.023 [Spearman's *p*-value]; Figure 3B), i.e., a relative increase in one group resulted in the decrease of the other group. Both of these groups are H_2_-utilizing methanogens, and presumably compete within the rumen [32]. Interestingly, there was a good positive correlation (R = 0.72, *P* = 6.2×10^–5^; Figure 4) between the occurrence of methanogens in the *Methanobrevibacter ruminantium* clade and bacteria in the family *Fibrobacteraceae*. In contrast, occurrence of methanogens in the *Methanobrevibacter gottschalkii* clade was positively correlated (R = 0.90, *P* = 4.9×10^–14^; Figure 4) with bacteria in the family *Ruminococcaceae*. *Ruminococcus* spp. and *Fibrobacter* spp. are both primary cellulose degraders in the rumen [49]. *Ruminococcus* spp. produce large amounts of H_2_, while *Fibrobacter* spp. produce formate [50]. Both H_2_ and formate are substrates for ruminal methanogens [51]. The co-variation of the two groups of methanogens with different bacteria suggests that the methanogens may be adapted to different ruminal H_2_ regimes or respond to differences in available CH_4_ precursors. *Methanobrevibacter ruminantium* M1, for example, appears to be specialized to low H_2_ concentrations based on the presence of only methyl coenzyme M reductase I (McrI) and the absence of the methyl coenzyme M reductase II (McrII) isoenzyme [52]. McrI and McrII are expressed at low- and high levels of H_2_, respectively, and members of the genus *Methanobrevibacter* usually contain both isoenzymes [53]. Genomes of more ruminal representatives of these methanogens are being sequenced, including members of the *Methanobrevibacter gottschalkii* clade [54]; the resulting genomic data may provide further insights into these occurrence patterns. Co-variation could also be the result of highly-specific interactions between certain methanogens and bacteria. It was recently shown that a bacterial flagellum protein induced a highly specific up-regulation of genes in methanogenesis [55], indicating that such targeted interactions between methanogens and bacteria may exist. Such interactions could facilitate a better transfer of H_2_ between the partners, benefitting both.

We also detected a strong positive correlation (R = 0.66, *P* = 2.5×10^–13^; Figure 4) between the occurrence of members of the archaeal Rumen Cluster C group (as defined by Janssen and Kirs [32]) and members of the methanogen genus *Methanosphaera*. This could indicate cooperation between the groups, or that they can coexist when their niche is expanded. *Methanosphaera* spp. and Rumen Cluster C archaea both produce CH_4_ from H_2_ plus methanol ([56]; J. Jeyanathan, R. S. Ronimus, S. O. Hoskin, P. H. Janssen, unpublished data). It seems likely that both groups proliferate when methanol availability is high, and that they somehow partition this niche, suggesting co-existence when a particular resource (methanol) is available.

As expected, co-occurrence analysis revealed a strong positive relationship between the rumen ciliate genera *Polyplastron* and *Ostracodinium* (R = 0.85, *P* = 1.8×10^–11^; Figure 4), two typical members of A-type ciliate communities in New Zealand ruminants [21]. Significant positive correlation was also observed between *Eudiplodinium*, a key genus of B-type ciliate communities and the Anoplodinium-Diplodinium cluster, species of which are commonly found in B-type ciliate communities (R = 0.76, *P* = 1.4×10^–8^; Figure 4). *Eudiplodinium* was also positively correlated with *Epidinium* (R = 0.58, *P* = 7.7×10^–8^; Figure 4), another key genus of B-type ciliate communities.

Significant correlations were further observed between certain ciliate protozoa and anaerobic fungi. Ciliates of the genus *Isotricha* appeared to be negatively correlated with anaerobic fungi of the BlackRhino group (R = −0.87, *P* = 8.1×10^–8^; Figure 4). In contrast, ciliate protozoa belonging to the genus *Entodinium* showed a strong positive relationship with Neocallimastix 1-group fungi (R = 0.81, *P* = 6.4×10^–8^; Figure 4).

## Conclusions

The approach we have described for analyzing the structure of bacterial, archaeal and eukaryotic microbial communities in the rumen ecosystem, in which all members of the community are simultaneously detected by large-scale pyrosequencing should have broad applications. It allows adjustments to accommodate differences in the abundance and diversity of different groups of microorganisms. Moreover, amplicons of different lengths can be combined by compensating for the different lengths, since shorter fragments appear to amplify preferentially. This type of study is not restricted to the analysis of small subunit ribosomal RNA genes, but can also be applied to investigate the environmental distribution of functional marker genes within the read length limit of available sequencing platforms.

Further investigation of the observed patterns of co-occurrence in the rumen may provide new clues about metabolic networks between rumen-inhabiting microbial groups and may resolve their individual contributions to overall rumen functioning. We also believe that looking beyond the bacteria and archaea and into the entirety of microbial communities, including eukaryotic microorganisms, will provide valuable new insights into the functioning of this and other microbial communities occupying diverse habitats.

## Supporting Information

Figure S1 **Rarefaction analysis of rumen microbial groups.**
(PDF)

Figure S2 **Comparison of primers for amplification of bacterial and archaeal 16S rRNA genes from rumen samples.**
(PDF)

Figure S3 **UPGMA cluster analysis of bacterial and archaeal communities analyzed with three different primer pairs each.**
(PDF)

Figure S4 **Lengths of obtained sequencing reads for two different mixing ratios of the microbial groups analyzed.**
(PDF)

Figure S5 **Bacterial community comparison of samples analyzed in two different sequencing runs using primer set BaL.**
(PDF)

Figure S6 **Bias of the three primer pairs targeting archaeal 16S rRNA genes.**
(PDF)

Figure S7 **Comparison of proportions of Bacteria and Archaea in rumen samples using pyrosequencing libraries and qPCR.**
(PDF)

Table S1 **Comparison of average relative abundances of fungal and ciliate groups across all rumen samples when pyrosequencing reads were analyzed using sequence databases sorted in alphabetical or in anti-alphabetical order by accession number.**
(PDF)

Table S2 **Ranges and averages of the numbers of phyla, classes, orders, and families detected across all DNA samples.**
(PDF)

Table S3 **Comparison of abundances of bacterial families detected by using 3 different primer sets (BaL, BaS, and ArBa) in all analyzed samples.**
(PDF)

Table S4 **Comparison of abundances of archaeal clades detected using 3 different primer sets (ArL, ArS, and ArBa) in all analyzed samples.**
(PDF)

Text S1 **Run-to-run variation.**
(PDF)

Text S2 **Diversity and coverage of bacterial and archaeal communities analyzed with different primer combinations.**
(PDF)

Text S3 **Estimation of the proportion of archaea obtained with the non-specific ArBa primer pair using qPCR and pyrosequencing libraries.**
(PDF)

## References

[1] Nollet L, Demeyer D, Verstraete W (1997) Effect of 2-bromoethanesulfonic acid and *Peptostreptococcus productus* ATCC 35244 addition on stimulation of reductive acetogenesis in the ruminal ecosystem by selective inhibition of methanogenesis. Applied & Environmental Microbiology 63: 194–200. 8979351 10.1128/aem.63.1.194-200.1997 PMC168314

[2] Smith P, Martino D, Cai Z, Gwary D, Janzen H, et al.. (2007) Agriculture. Contribution of Working Group III to the Fourth Assessment Repirt of the IPCC ed. Cambridge: Cambridge University Press.

[3] Buddle BM, Denis M, Attwood GT, Altermann E, Janssen PH, et al (2011) Strategies to reduce methane emissions from farmed ruminants grazing on pasture. Veterinary Journal 188: 11–17. 10.1016/j.tvjl.2010.02.019 20347354

[4] Akin DE, Benner R (1988) Degradation of polysaccharides and lignin by ruminal bacteria and fungi. Applied & Environmental Microbiology 54: 1117–1125. 3389808 10.1128/aem.54.5.1117-1125.1988 PMC202614

[5] Bauchop T, Clarke RTJ (1976) Attachment of the ciliate *Epidinium* to plant fragments in the sheep rumen. Applied & Environmental Microbiology 32: 417–422. 825041 10.1128/aem.32.3.417-422.1976 PMC170080

[6] Brulc JM, Antonopoulos DA, Miller MEB, Wilson MK, Yannarell AC, et al (2009) Gene-centric metagenomics of the fiber-adherent bovine rumen microbiome reveals forage specific glycoside hydrolases. Proceedings of the National Academy of Sciences of the United States of America 106: 1948–1953. 19181843 10.1073/pnas.0806191105 PMC2633212

[7] Callaway TR, Dowd SE, Edrington TS, Anderson RC, Krueger N, et al (2010) Evaluation of bacterial diversity in the rumen and feces of cattle fed different levels of dried distillers grains plus solubles using bacterial tag-encoded FLX amplicon pyrosequencing. Journal of Animal Science 88: 3977–3983. 20729286 10.2527/jas.2010-2900

[8] Pitta DW, Pinchak WE, Dowd SE, Osterstock J, Gontcharova V, et al (2010) Rumen bacterial diversity dynamics associated with changing from Bermudagrass hay to grazed winter wheat diets. Microbial Ecology 59: 511–522. 20037795 10.1007/s00248-009-9609-6

[9] Williams WL, Tedeschi LO, Kononoff PJ, Callaway TR, Dowd SE, et al (2010) Evaluation of *in vitro* gas production and rumen bacterial populations fermenting corn milling (co)products. Journal of Dairy Science 93: 4735–4743. 20855008 10.3168/jds.2009-2920

[10] Li RW, Connor EE, Li C, Baldwin RLV, Sparks ME (2012) Characterization of the rumen microbiota of pre-ruminant calves using metagenomic tools. Environmental Microbiology 14: 129–139. 21906219 10.1111/j.1462-2920.2011.02543.x

[11] Hess M, Sczyrba A, Egan R, Kim T-W, Chokhawala H, et al (2011) Metagenomic discovery of biomass-degrading genes and genomes from cow rumen. Science 331: 463–467. 21273488 10.1126/science.1200387

[12] Jami E, Mizrahi I (2012) Composition and similarity of bovine rumen microbiota across individual animals. PLoS ONE 7: e33306. 22432013 10.1371/journal.pone.0033306 PMC3303817

[13] Wu S, Baldwin RLV, Li W, Li C, Connor EE, et al.. (2012) The bacterial community composition of the bovine rumen detected using pyrosequencing of 16S rRNA genes. Metagenomics 1: Article ID 235571.

[14] Parfrey LW, Walters WA, Knight R (2011) Microbial eukaryotes in the human microbiome: ecology, evolution, and future directions. Frontiers in Microbiology 2: 153. 21808637 10.3389/fmicb.2011.00153 PMC3135866

[15] Douglas AE (1994) Symbiotic interactions. Symbiotic interactions. Oxford, UK: Oxford University Press. 148.

[16] Bootten TJ, Joblin KN, McArdle BH, Harris PJ (2011) Degradation of lignified secondary cell walls of lucerne (*Medicago sativa* L.) by rumen fungi growing in methanogenic co-culture. Journal of Applied Microbiology 111: 1086–1096. 21848807 10.1111/j.1365-2672.2011.05127.x

[17] Dehority BA, Odenyo AA (2003) Influence of diet on the rumen protozoal fauna of indigenous African wild ruminants. Journal of Eukaryotic Microbiology 50: 220–223. 12836880 10.1111/j.1550-7408.2003.tb00121.x

[18] Chen Y-C, Tsai S-D, Cheng H-L, Chien C-Y, Hu C-Y, et al (2007) *Caecomyces sympodialis* sp. nov., a new rumen fungus isolated from *Bos indicus* . Mycologia 99: 125–130. 17663130 10.3852/mycologia.99.1.125

[19] Dehority BA (2010) Physiological characteristics of several rumen protozoa grown *in vitro* with observations on within and among species variation. European Journal of Protistology 46: 271–279. 20801008 10.1016/j.ejop.2010.05.002

[20] Liggenstoffer AS, Youssef NH, Couger MB, Elshahed MS (2010) Phylogenetic diversity and community structure of anaerobic gut fungi (phylum *Neocallimastigomycota*) in ruminant and non-ruminant herbivores. ISME Journal 4: 1225–1235. 20410935 10.1038/ismej.2010.49

[21] Kittelmann S, Janssen PH (2011) Characterization of rumen ciliate community composition in domestic sheep, deer, and cattle, feeding on varying diets, by means of PCR-DGGE and clone libraries. FEMS Microbiology Ecology 75: 468–481. 21204869 10.1111/j.1574-6941.2010.01022.x

[22] Kittelmann S, Naylor GE, Koolaard JP, Janssen PH (2012) A proposed taxonomy of anaerobic fungi (Class *Neocallimastigomycetes*) suitable for large-scale sequence-based community structure analysis. PLoS ONE 7: Article No. e36866. 10.1371/journal.pone.0036866 PMC3353986 22615827

[23] Jeyanathan J, Kirs M, Ronimus RS, Hoskin SO, Janssen PH (2011) Methanogen community structure in the rumens of farmed sheep, cattle and red deer fed different diets. FEMS Microbiology Ecology 76: 311–326. 21255054 10.1111/j.1574-6941.2011.01056.x

[24] Ramirez-Restrepo CA, Barry TN, Marriner A, Lopez-Villalobos N, McWilliam EL, et al (2010) Effects of grazing willow fodder blocks upon methane production and blood composition in young sheep. Animal Feed Science & Technology 155: 33–43.

[25] Lueders T, Manefield M, Friedrich MW (2004) Enhanced sensitivity of DNA- and rRNA-based stable isotope probing by fractionation and quantitative analysis of isopycnic centrifugation gradients. Environmental Microbiology 6: 73–78. 14686943 10.1046/j.1462-2920.2003.00536.x

[26] Caporaso JG, Lauber CL, Walters WA, Berg-Lyons D, Lozupone CA, et al (2011) Global patterns of 16S rRNA diversity at a depth of millions of sequences per sample. Proceedings of the National Academy of Sciences of the United States of America 108: 4516–4522. 20534432 10.1073/pnas.1000080107 PMC3063599

[27] Fierer N, Hamady M, Lauber CL, Knight R (2008) The influence of sex, handedness, and washing on the diversity of hand surface bacteria. Proceedings of the National Academy of Sciences of the United States of America 105: 17994–17999. 19004758 10.1073/pnas.0807920105 PMC2584711

[28] Walters WA, Caporaso JG, Lauber CL, Berg-Lyons D, Fierer N, et al (2011) PrimerProspector: de novo design and taxonomic analysis of barcoded polymerase chain reaction primers. Bioinformatics 27: 1159–1161. 21349862 10.1093/bioinformatics/btr087 PMC3072552

[29] Caporaso JG, Kuczynski J, Stombaugh J, Bittinger K, Bushman FD, et al (2010) QIIME allows analysis of high-throughput community sequencing data. Nature Methods 7: 335–336. 20383131 10.1038/nmeth.f.303 PMC3156573

[30] Edgar RC (2010) Search and clustering orders of magnitude faster than BLAST. Bioinformatics 26: 2460–2461. 20709691 10.1093/bioinformatics/btq461

[31] McDonald D, Price MN, Goodrich J, Nawrocki EP, DeSantis TZ, et al (2011) An improved Greengenes taxonomy with explicit ranks for ecological and evolutionary analyses of bacteria and archaea. ISME Journal 6: 610–618. 22134646 10.1038/ismej.2011.139 PMC3280142

[32] Janssen PH, Kirs M (2008) Structure of the archaeal community of the rumen. Applied & Environmental Microbiology 74: 3619–3625. 18424540 10.1128/AEM.02812-07 PMC2446570

[33] Goodman R (1957) Teach yourself statistics. London, UK: English Universities Press. 240 p.

[34] Stackebrandt E, Goebel BM (1994) Taxonomic note: A place for DNA-DNA reassociation and 16S rRNA sequence analysis in the present species definition in bacteriology. International Journal of Systematic Bacteriology 44: 846–849.

[35] Simpson EH (1949) Measurement of diversity. Nature Methods 163: 688.

[36] Hammer O, Harper DAT, Ryan PD (2001) PAST: Palaeontological statistics software package for education and data analysis. Palaeontologia Electronica 4: 1–9.

[37] Ihaka R, Gentlemen R (1996) R: a language for data analysis and graphics. Journal of Computational and Graphical Statistics 5: 299–314.

[38] Murdoch DJ, Chow ED (1996) A graphical display of large correlation matrices. The American Statistician 50: 178–180.

[39] Harrell FEJ (2012) Package ‘Hmisc’ – Harrell Miscellaneous. http://cranr-projectorg/web/packages/Hmisc/Hmiscpdf: 1–362.

[40] Wei T (2012) Package ‘corrplot’ – Visualization of a correlation matrix v0.60. http://cranr-projectorg/web/packages/corrplot/corrplotpdf: 1–16.

[41] RocheDiagnostics (2011) Technical Bulletin – GS FLX System – Short Fragment Removal Procedure. http://uagc.arl.arizona.edu/sites/default/files/u4/454ShortFragmentRemoval Procedure_Original.pdf, TCB No. 2011–002: 1–8.

[42] Amaral-Zettler LA, Zettler ER, Theroux SM, Palacios C, Aguilera A, et al (2011) Microbial community structure across the tree of life in the extreme Rio Tinto. ISME Journal 5: 42–50. 20631808 10.1038/ismej.2010.101 PMC3105667

[43] Sinigalliano CD, Gidley ML, Shibata T, Whitman D, Dixon TH, et al (2007) Impact of hurricanes Katrina and Rita on the microbial landscape of the New Orleans area. Proceedings of the National Academy of Sciences of the United States of America 104: 9029–9034. 17488814 10.1073/pnas.0610552104 PMC1885622

[44] Rowe JB, Loughnan ML, Nolan JV, Leng RA (1979) Secondary fermentation in the rumen of a sheep given a diet based on molasses. British Journal of Nutrition 41: 393–396. 427091 10.1079/bjn19790048

[45] Domingue BMF, Dellow DW, Wilson PR, Barry TN (1991) Comparative Digestion in Deer Goats and Sheep. New Zealand Journal of Agricultural Research 34: 45–54.

[46] Dehority BA, Orpin CG (1997) Development of, and natural fluctuations in rumen microbial populations. In: Hobson PN, Stewart CS, editors. The rumen microbial ecosystem. New York: Blackie Academic & Professional. 196–235.

[47] Eadie JM (1962) Inter-relationships between certain rumen ciliate protozoa. Journal of General Microbiology 29: 579–588.

[48] Sundset MA, Edwards JE, Cheng YF, Senosiain RS, Fraile MN, et al (2009) Molecular diversity of the rumen microbiome of Norwegian reindeer on natural summer pasture. Microbial Ecology 57: 335–348. 18604648 10.1007/s00248-008-9414-7

[49] Kobayashi Y, Shinkai T, Koike S (2008) Ecological and physiological characterization shows that *Fibrobacter succinogenes* is important in rumen fiber digestion – Review. Folia Microbiologica 53: 195–200. 18661290 10.1007/s12223-008-0024-z

[50] Rychlik JL, May T (2000) The effect of a methanogen, *Methanobrevibacter smithii*, on the growth rate, organic acid production, and specific ATP activity of three predominant ruminal cellulolytic bacteria. Current Microbiology 40: 176–180. 10679049 10.1007/s002849910035

[51] Balch WE, Fox GE, Magrum LJ, Woese CR, Wolfe RS (1979) Methanogens: Reevaluation of a unique biological group. Microbiological Reviews 43: 260–296. 390357 10.1128/mr.43.2.260-296.1979 PMC281474

[52] Leahy SC, Kelly WJ, Altermann E, Ronimus RS, Yeoman CJ, et al. (2010) The genome sequence of the rumen methanogen *Methanobrevibacter ruminantium* reveals new possibilities for controlling ruminant methane emissions. PLoS ONE 5: Article No. e8926. 10.1371/journal.pone.0008926 PMC2812497 20126622

[53] Reeve JN, Noelling J, Morgan RM, Smith DR (1997) Methanogenesis: Genes, genomes, and who's on first? Journal of Bacteriology 179: 5975–5986. 9324240 10.1128/jb.179.19.5975-5986.1997 PMC179496

[54] Morgavi D, Kelly WJ, Janssen PH, Attwood GT (in press) Rumen microbial (meta)genomics and its application to ruminant production. Animal, doi:10.1017/S1751731112000419. 10.1017/S1751731112000419 23031271

[55] Shimoyama T, Kato S, Ishii Si, Watanabe K (2009) Flagellum mediates symbiosis. Science 323: 1574. 19299611 10.1126/science.1170086

[56] Fricke WF, Seedorf H, Henne A, Krueer M, Liesegang H, et al (2006) The genome sequence of *Methanosphaera stadtmanae* reveals why this human intestinal archaeon is restricted to methanol and H_2_ for methane formation and ATP synthesis. Journal of Bacteriology 188: 642–658. 16385054 10.1128/JB.188.2.642-658.2006 PMC1347301

[57] Takai K, Horikoshi K (2000) Rapid detection and quantification of members of the archaeal community by quantitative PCR using fluorogenic probes. Applied & Environmental Microbiology 66: 5066–5072. 11055964 10.1128/aem.66.11.5066-5072.2000 PMC92420

[58] Weisburg WG, Barns SM, Pelletier DA, Lane DJ (1991) 16S ribosomal DNA amplification for phylogenetic study. Journal of Bacteriology 173: 697–703. 1987160 10.1128/jb.173.2.697-703.1991 PMC207061

[59] Lane DJ, Pace B, Olsen GJ, Stahl DA, Sogin ML, et al (1985) Rapid determination of 16S ribosomal RNA sequences for phylogenetic analyses. Proceedings of the National Academy of Sciences of the United States of America 82: 6955–6959. 2413450 10.1073/pnas.82.20.6955 PMC391288

[60] Edwards U, Rogall T, Bloecker H, Emde M, Boettger EC (1989) Isolation and direct complete nucleotide determination of entire genes. Characterization of a gene coding for 16S ribosomal RNA. Nucleic Acids Research 17: 7843–7854. 2798131 10.1093/nar/17.19.7843 PMC334891

[61] Amann RI, Ludwig W, Schleifer K-H (1995) Phylogenetic identification and *in situ* detection of individual microbial cells without cultivation. Microbiological Reviews 59: 143–169. 7535888 10.1128/mr.59.1.143-169.1995 PMC239358

[62] Watanabe T, Asakawa S, Nakamura A, Nagaoka K, Kimura M (2004) DGGE method for analyzing 16S rDNA of methanogenic archaeal community in paddy field soil. FEMS Microbiology Letters 232: 153–163. 15033234 10.1016/S0378-1097(04)00045-X

[63] Skillman LC, Evans PN, Naylor GE, Morvan B, Jarvis GN, et al (2004) 16S ribosomal DNA-directed PCR primers for ruminal methanogens and identification of methanogens colonising young lambs. Anaerobe 10: 277–285. 16701528 10.1016/j.anaerobe.2004.05.003

[64] Casamayor EO, Massana R, Benlloch S, Ovreas L, Diez B, et al (2002) Changes in archaeal, bacterial and eukaryal assemblages along a salinity gradient by comparison of genetic fingerprinting methods in a multipond solar saltern. Environmental Microbiology 4: 338–348. 12071979 10.1046/j.1462-2920.2002.00297.x

[65] Ovreas L, Forney L, Daae FL, Torsvik V (1997) Distribution of bacterioplankton in meromictic Lake Saelenvannet, as determined by denaturing gradient gel electrophoresis of PCR-amplified gene fragments coding for 16SrRNA. Applied & Environmental Microbiology 63: 3367–3373. 9292986 10.1128/aem.63.9.3367-3373.1997 PMC168642

[66] Regensbogenova M, Pristas P, Javorsky P, Moon-van der Staay SY, van der Staay GWM, et al (2004) Assessment of ciliates in the sheep rumen by DGGE. Letters in Applied Microbiology 39: 144–147. 15242452 10.1111/j.1472-765X.2004.01542.x

[67] Tuckwell DS, Nicholson MJ, McSweeney CS, Theodoro MK, Brookman JL (2005) The rapid assignment of ruminal fungi to presumptive genera using ITS1 and ITS2 RNA secondary structures to produce group-specific fingerprints. Microbiology 151: 1557–1567. 15870465 10.1099/mic.0.27689-0

