# Supplementary material for: Simultaneous Amplicon Sequencing to Explore Co-Occurrence Patterns of Bacterial, Archaeal and Eukaryotic Microorganisms in Rumen Microbial Communities
Source: PLoS One. 2013 Feb 8;8(2):e47879. doi: 10.1371/journal.pone.0047879 (PMC3568148; doi:10.1371/journal.pone.0047879)

Figure S1. Rarefaction analysis of rumen microbial groups. Collector's curves describing (A) the number of bacterial (□), archaeal (Δ), ciliate (◊), and anaerobic fungal (○) OTUs detected per sequence sampled at sequence similarity levels of 97% (black) and 95% (white), and (B) the percentage of novel bacterial (□), archaeal (Δ), ciliate (◊), and anaerobic fungal (○) OTUs detected per sequence sampled at sequence similarity levels of 97% (black fill) and 95% (white fill). Curves represent the averages of the microbial communities in the 12 pyrosequencing libraries.

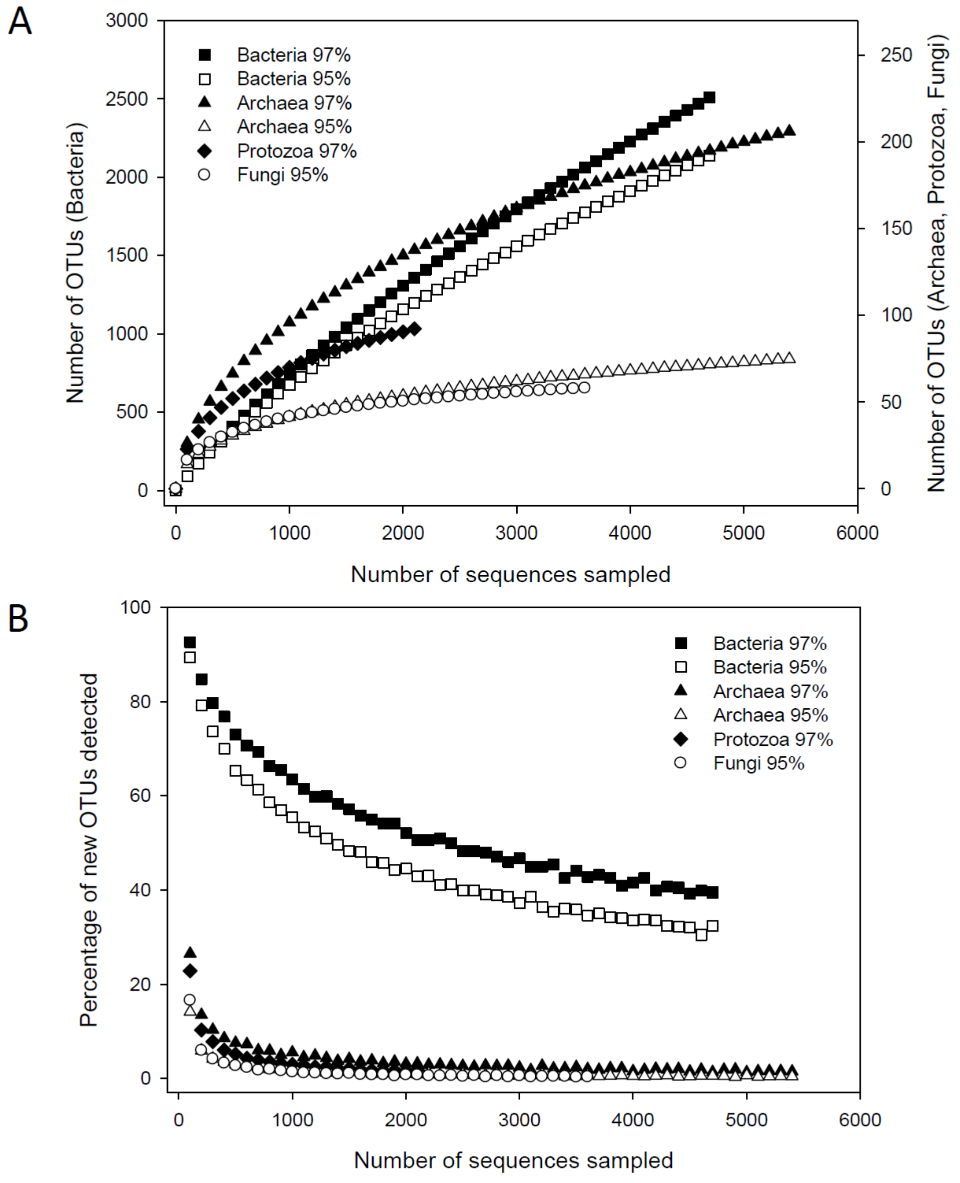

Supplement: Figure S1 — Rarefaction analysis of rumen microbial groups. (PDF) [file pone.0047879.s001.pdf]
