# Supplementary material for: Simultaneous Amplicon Sequencing to Explore Co-Occurrence Patterns of Bacterial, Archaeal and Eukaryotic Microorganisms in Rumen Microbial Communities
Source: PLoS One. 2013 Feb 8;8(2):e47879. doi: 10.1371/journal.pone.0047879 (PMC3568148; doi:10.1371/journal.pone.0047879)

Figure S2. Comparison of primers for amplification of bacterial and archaeal 16S rRNA genes from rumen samples. Two-dimensional principal coordinates diagrams showing the similarity of (A) bacterial communities coloured by primer sequence, (B) bacterial communities coloured by sample identity, (C) archaeal communities coloured by primer sequence, and (D) archaeal communities coloured by sample identity of the 12 DNA samples analyzed. Primer pairs: BaL/ArL = green, BaS/ArS = red, ArBa = blue. Sample identities: C5SGPN = light blue circles, C5SIPN = turquoise sideways triangles, C6HYPN = dark blue sideways triangles, C7PATK = dark blue pentagon, D3WGPN = dark green triangles, D4SGPN = light green triangles, S19PAPN = orange squares, S2SIPN = dark purple diamonds, S4SG1PN = pink squares, S4SG2PN = pink circles, S4WGPN = red hexagon, S5GRRK = yellow triangles.

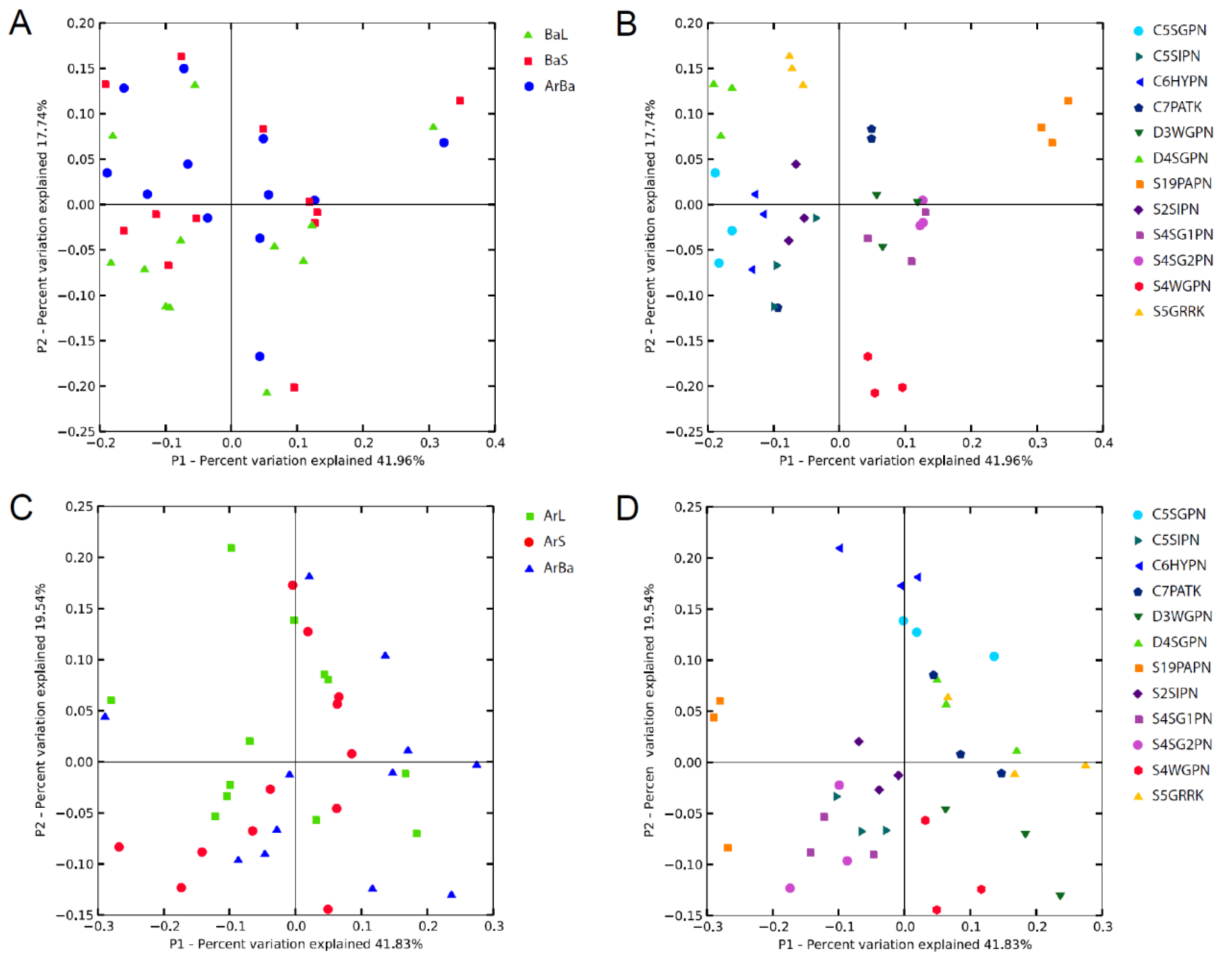

Supplement: Figure S2 — Comparison of primers for amplification of bacterial and archaeal 16S rRNA genes from rumen samples. (PDF) [file pone.0047879.s002.pdf]
