# Supplementary material for: Simultaneous Amplicon Sequencing to Explore Co-Occurrence Patterns of Bacterial, Archaeal and Eukaryotic Microorganisms in Rumen Microbial Communities
Source: PLoS One. 2013 Feb 8;8(2):e47879. doi: 10.1371/journal.pone.0047879 (PMC3568148; doi:10.1371/journal.pone.0047879)

Figure S3. UPGMA cluster analysis of bacterial and archaeal communities analyzed with three different primer pairs each. UPGMA clustering was performed based on Bray-Curtis dissimilarity matrices of the (A) bacterial and (B) archaeal communities in 12 DNA samples amplified with the primer pairs BaL, BaS, and ArBa or ArL, ArS, and ArBa, respectively. The scale bar represents 5% difference in community structure. Libraries are colour-coded by sample ID.

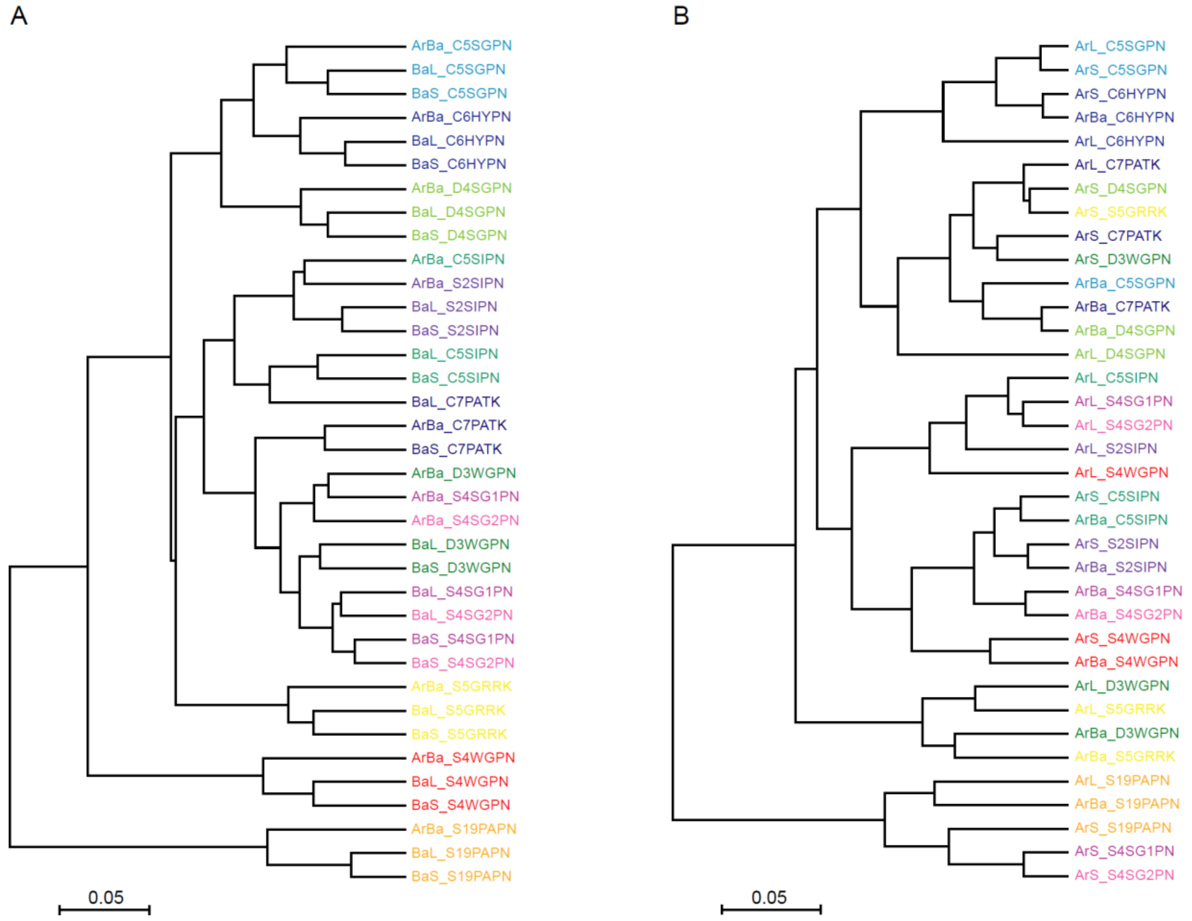

Supplement: Figure S3 — UPGMA cluster analysis of bacterial and archaeal communities analyzed with three different primer pairs each. (PDF) [file pone.0047879.s003.pdf]
