# Supplementary material for: Simultaneous Amplicon Sequencing to Explore Co-Occurrence Patterns of Bacterial, Archaeal and Eukaryotic Microorganisms in Rumen Microbial Communities
Source: PLoS One. 2013 Feb 8;8(2):e47879. doi: 10.1371/journal.pone.0047879 (PMC3568148; doi:10.1371/journal.pone.0047879)

Figure S4. Lengths of obtained sequencing reads for two different mixing ratios of the microbial groups analyzed. Distribution of barcode mappable reads according to fragment length after quality control for the mixing ratios of 5:1:1:1 (open circles) and 5:1:1:0.2 (filled circles). The mixing ratios represent bacteria:archaea:ciliate protozoa:fungi.

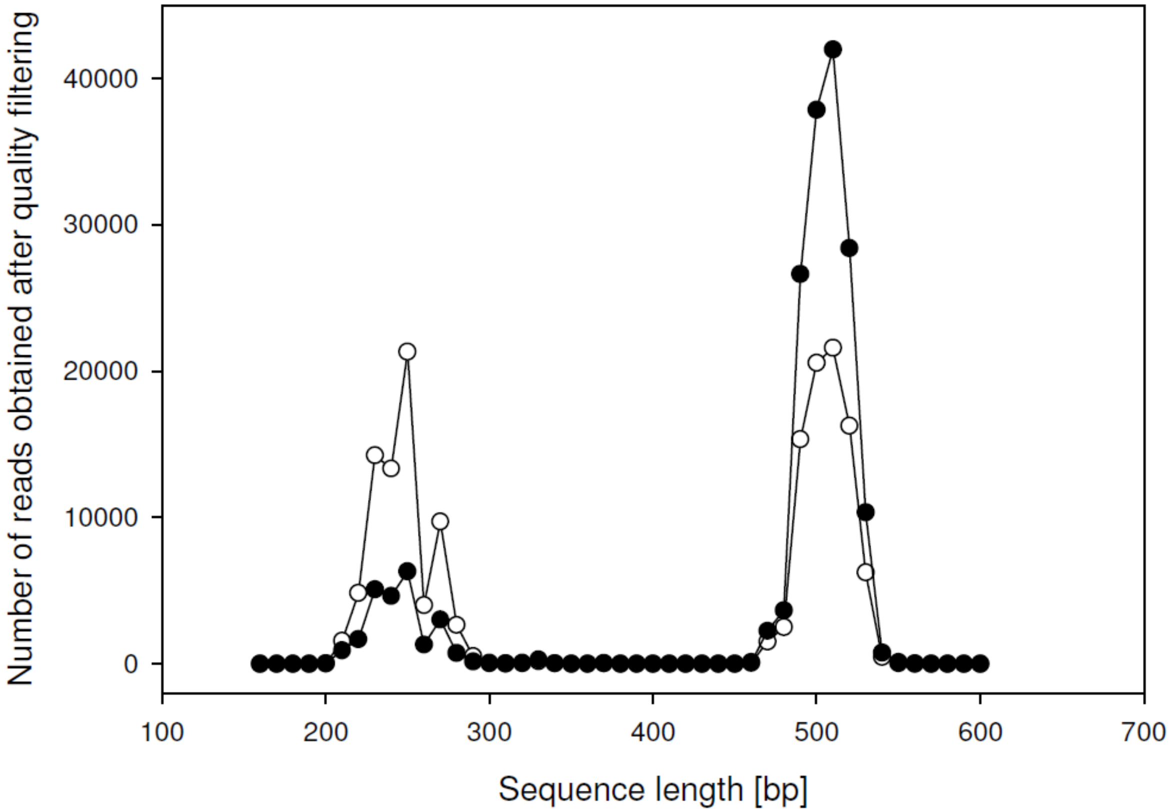

Supplement: Figure S4 — Lengths of obtained sequencing reads for two different mixing ratios of the microbial groups analyzed. (PDF) [file pone.0047879.s004.pdf]
