# Supplementary material for: Simultaneous Amplicon Sequencing to Explore Co-Occurrence Patterns of Bacterial, Archaeal and Eukaryotic Microorganisms in Rumen Microbial Communities
Source: PLoS One. 2013 Feb 8;8(2):e47879. doi: 10.1371/journal.pone.0047879 (PMC3568148; doi:10.1371/journal.pone.0047879)

Figure S5. Bacterial community comparison of samples analyzed in two different sequencing runs using primer set BaL. UPGMA cluster analysis of the bacterial communities obtained from 12 DNA samples amplified with the BaL primer pair and sequenced in two different sequencing runs (prefixes BaL_1 or BaL_2). Bray-Curtis was used as dissimilarity distance metric. The scale bar represents a dissimilarity of 5%. The sample identifiers are given in Table 1.

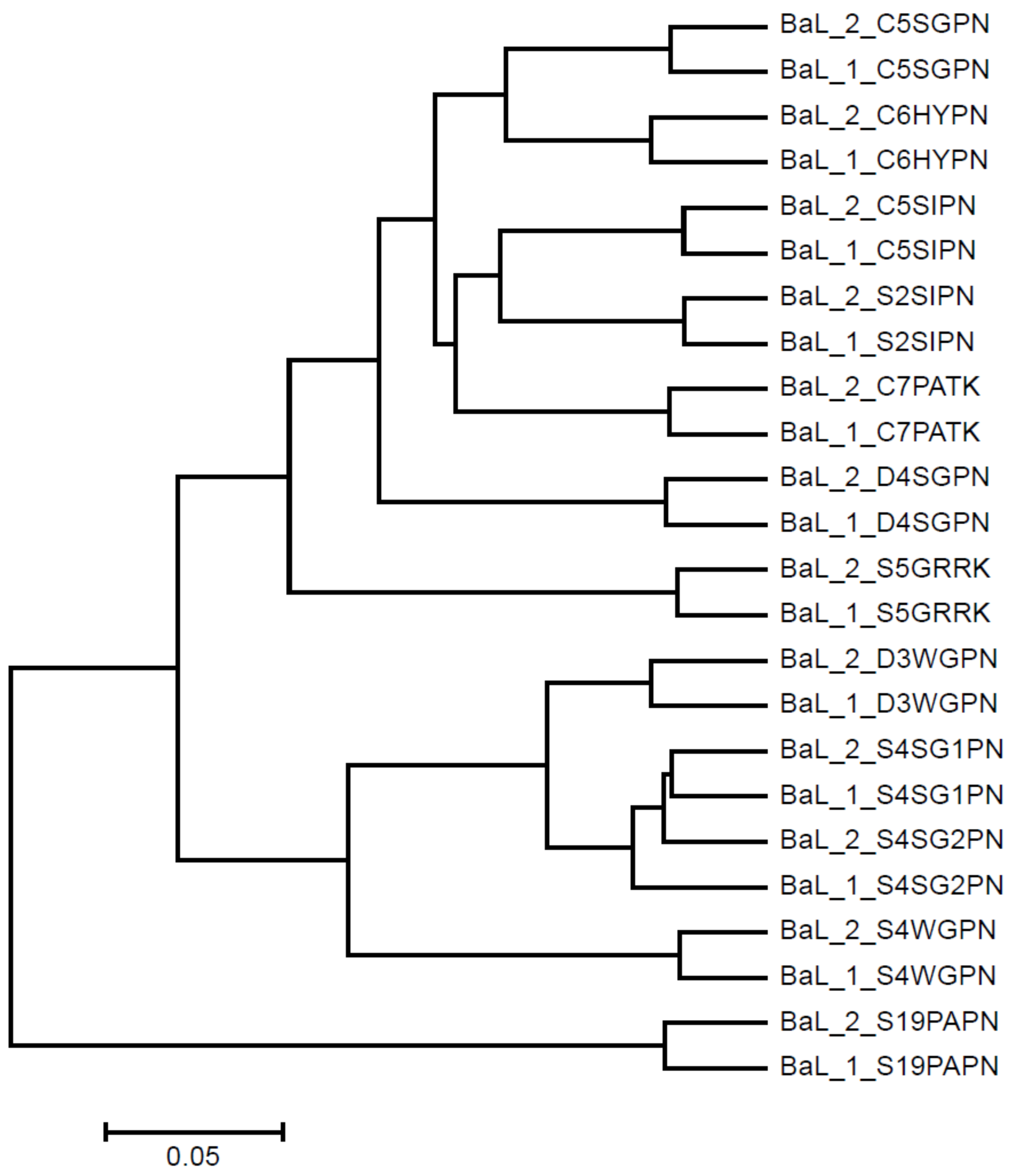

Supplement: Figure S5 — Bacterial community comparison of samples analyzed in two different sequencing runs using primer set BaL. (PDF) [file pone.0047879.s005.pdf]
