# Supplementary material for: Simultaneous Amplicon Sequencing to Explore Co-Occurrence Patterns of Bacterial, Archaeal and Eukaryotic Microorganisms in Rumen Microbial Communities
Source: PLoS One. 2013 Feb 8;8(2):e47879. doi: 10.1371/journal.pone.0047879 (PMC3568148; doi:10.1371/journal.pone.0047879)

Figure S6. Bias of the three primer pairs targeting archaeal 16S rRNA genes. Comparison of primer sets targeting archaeal 16S rRNA genes based on the relative abundance of the four dominant archaeal groups detected in this study. Dashed lines indicate no significant differences between any two primer pairs in the abundance of a certain clade of methanogens. Solid lines indicate significant differences in abundances with the *P*-values given in brackets and the arrows pointing towards the primer pair giving a significantly higher abundance. Major methanogen groups are abbreviated as follows: Mbbr = *Methanobrevibacter ruminantium* and relatives, Mbbg = *Methanobrevibacter gottschalkii* and relatives, Msp = *Methanosphaera*, RCC = Rumen Cluster C.

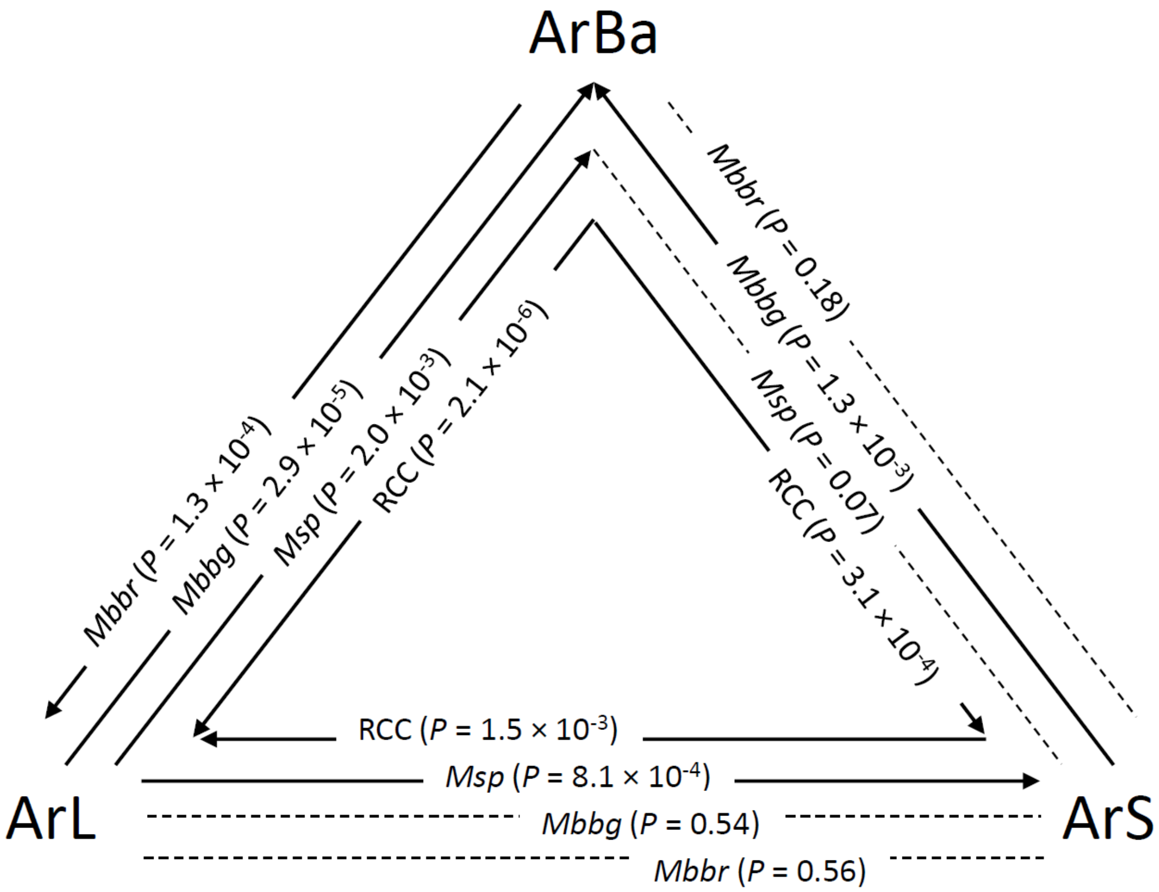

Supplement: Figure S6 — Bias of the three primer pairs targeting archaeal 16S rRNA genes. (PDF) [file pone.0047879.s006.pdf]
