# Supplementary material for: Simultaneous Amplicon Sequencing to Explore Co-Occurrence Patterns of Bacterial, Archaeal and Eukaryotic Microorganisms in Rumen Microbial Communities
Source: PLoS One. 2013 Feb 8;8(2):e47879. doi: 10.1371/journal.pone.0047879 (PMC3568148; doi:10.1371/journal.pone.0047879)

Figure S7. Comparison of proportions of Bacteria and Archaea in rumen samples using pyrosequencing libraries and qPCR. (A) Relative abundances of bacterial (Δ) and archaeal (○) 16S rRNA genes based on relative read numbers obtained from pyrosequencing libraries constructed with the ArBa primer pair (open symbols) or via qPCR (filled symbols) for the 12 DNA samples analyzed, and (B) correlation between relative abundance of archaeal 16S rRNA genes as determined via pyrosequencing libraries and qPCR.

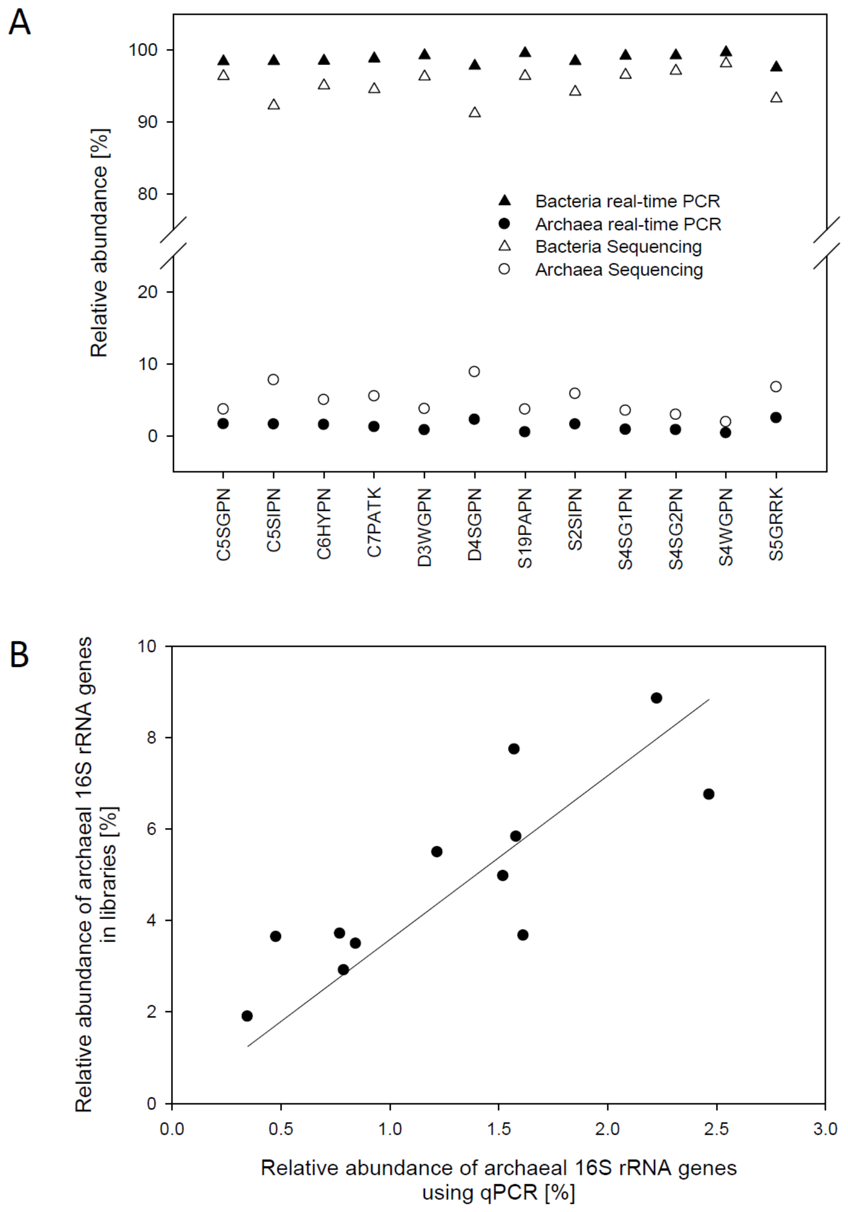

Supplement: Figure S7 — Comparison of proportions of Bacteria and Archaea in rumen samples using pyrosequencing libraries and qPCR. (PDF) [file pone.0047879.s007.pdf]
