# Supplementary material for: Simultaneous Amplicon Sequencing to Explore Co-Occurrence Patterns of Bacterial, Archaeal and Eukaryotic Microorganisms in Rumen Microbial Communities
Source: PLoS One. 2013 Feb 8;8(2):e47879. doi: 10.1371/journal.pone.0047879 (PMC3568148; doi:10.1371/journal.pone.0047879)
Supplement: Table S1 — Comparison of average relative abundances of fungal and ciliate groups across all rumen samples when pyrosequencing reads were analyzed using sequence databases sorted in alphabetical or in anti-alphabetical order by accession number. (PDF) [file pone.0047879.s008.pdf]

Table S1. Comparison of average relative abundances of fungal and ciliate groups across all rumen samples when pyrosequencing reads were analyzed using sequence databases sorted in alphabetical or in anti-alphabetical order by accession number. The table reports the discrepancies between using the two differently sorted sequence files as reference databases, e.g., 0.0068 % ± 0.015 % of all sequences in the BlackRhino group did not cluster into this group reproducibly with both methods. Taxonomic groups of anaerobic fungi and ciliate protozoa that were detected in the 12 analyzed pyrosequencing libraries but did not show any differences in abundance when using the two different methods are not listed.

**Microbial group**	**Clade**	**Average difference ± Standard deviation**
Anaerobic fungi	BlackRhino	$6.8 \times 10^{-5} \pm 1.5 \times 10^{-4}$
	Caecomyces 1	$1.5 \times 10^{-4} \pm 1.8 \times 10^{-4}$
	Cyllamyces 2	$7.9 \times 10^{-5} \pm 2.7 \times 10^{-4}$
	Neocallimastix 1	$7.3 \times 10^{-6} \pm 2.5 \times 10^{-5}$
	Orpinomyces 5	$3.1 \times 10^{-5} \pm 7.8 \times 10^{-5}$
	Piromyces 2	$3.8 \times 10^{-4} \pm 1.2 \times 10^{-3}$
	Piromyces 3	$2.1 \times 10^{-5} \pm 4.8 \times 10^{-5}$
	Piromyces 7	$4.9 \times 10^{-4} \pm 1.3 \times 10^{-3}$
	SK3	$5.8 \times 10^{-5} \pm 1.5 \times 10^{-4}$

	SK4	$3.4 \times 10^{-6} \pm 1.2 \times 10^{-5}$
Ciliate protozoa	Anoplod.-Diplod.	$6.96 \times 10^{-5} \pm 1.27 \times 10^{-4}$
	Enoploplastron	$6.11 \times 10^{-4} \pm 1.75 \times 10^{-3}$
	Entodinium	$2.40 \times 10^{-4} \pm 3.79 \times 10^{-4}$
	Epidinium	$1.67 \times 10^{-4} \pm 2.39 \times 10^{-4}$
	Eremopl.-Diplopl.	$7.70 \times 10^{-3} \pm 9.77 \times 10^{-3}$
	Eudiplodinium	$1.70 \times 10^{-3} \pm 2.30 \times 10^{-3}$
	Metadinium	$9.48 \times 10^{-3} \pm 1.21 \times 10^{-2}$
	Ostracodinium	$1.17 \times 10^{-4} \pm 1.83 \times 10^{-4}$
	Polyplastron	$1.93 \times 10^{-4} \pm 4.56 \times 10^{-4}$
