# Supplementary material for: Simultaneous Amplicon Sequencing to Explore Co-Occurrence Patterns of Bacterial, Archaeal and Eukaryotic Microorganisms in Rumen Microbial Communities
Source: PLoS One. 2013 Feb 8;8(2):e47879. doi: 10.1371/journal.pone.0047879 (PMC3568148; doi:10.1371/journal.pone.0047879)
Supplement: Table S2 — Ranges and averages of the numbers of phyla, classes, orders, and families detected across all DNA samples. (PDF) [file pone.0047879.s009.pdf]

Table S2. Ranges and averages of the numbers of phyla, classes, orders, and families detected across all DNA samples when using different primer sets targeting the Bacteria and Archaea. Comparison of primer pairs targeting Bacteria (BaL, BaS, and ArBa) and methanogenic archaea (ArL, ArS, and ArBa) in regards to diversity at phylum-, class-, order-, and family-level.

		**BaL**	**BaS**	**ArBa**	**ArL**	**ArS**	**ArBa**
No. of phyla	Min	6	6	7	1	1	1
	Max	11	10	11	2	1	1
	Mean	8.8	8.6	9.1	1.8	1	1
No. of classes	Min	7	7	7	2	2	2
	Max	12	12	12	4	3	3
	Mean	9.7	9.7	9.1	3.3	2.3	2.3
No. of orders	Min	7	7	7	2	2	2
	Max	12	12	12	5	4	3
	Mean	9.5	9.8	8.9	3.8	2.5	2.3
No. of families	Min	13	13	12	2	2	2
	Max	18	19	18	5	4	3
	Mean	15.1	15.7	14.8	3.8	2.5	2.3
