# Supplementary material for: Simultaneous Amplicon Sequencing to Explore Co-Occurrence Patterns of Bacterial, Archaeal and Eukaryotic Microorganisms in Rumen Microbial Communities
Source: PLoS One. 2013 Feb 8;8(2):e47879. doi: 10.1371/journal.pone.0047879 (PMC3568148; doi:10.1371/journal.pone.0047879)
Supplement: Table S3 — Comparison of abundances of bacterial families detected by using 3 different primer sets (BaL, BaS, and ArBa) in all analyzed samples. (PDF) [file pone.0047879.s010.pdf]

Table S3. Comparison of abundances of bacterial families detected by using 3 different primer sets (BaL, BaS, and ArBa) in all analyzed samples. Comparison of primer pairs targeting Bacteria in regards to their coverage at family level. Families are ranked according to their abundances using primer pair BaL, and added up in that order for primer pairs BaS and ArBa to give a cumulative total (Sum).

**Phylum, Family**	**BaL**			**BaS**			**ArBa**		
	**Rank**	**Abundance**	**Sum**	**Rank**	**Abundance**	**Sum**	**Rank**	**Abundance**	**Sum**
		**[%]**	**[%]**		**[%]**	**[%]**		**[%]**	**[%]**
Firmicutes, Lachnospiraceae	1	24.1	24.1	2	19.7	19.7	2	17.8	17.8
Bacteroidetes, Prevotellaceae	2	23.8	47.9	1	26.1	45.8	1	24.1	41.9
Bacteroidetes, Other	3	14.3	62.2	3	13.8	59.6	3	12.9	54.8
Firmicutes, Other	4	8.2	70.4	4	9.9	69.5	4	11.5	66.3
Firmicutes, Ruminococcaceae	5	8.1	78.5	5	8.4	77.5	5	9.4	75.7
Fibrobacteraceae	6	5.3	83.8	6	5.0	82.9	6	5.3	81.0
Cyanobacteria, Other	7	1.7	85.5	10	1.5	84.4	21	0.2	81.2

Firmicutes, Veillonellaceae	8	1.5	87.0	7	2.3	86.7	8	3.3	84.5
Spirochaetaceae	9	1.3	88.3	8	1.7	88.4	7	4.6	89.1
Firmicutes, Clostridiales Family XIII.	10	1.3	89.6	21	0.4	88.8	9	1.3	90.4
Bacteroidetes, Porphyromonadaceae	11	1.3	90.9	9	1.6	90.4	10	1.2	91.6
Actinobacteria, Coriobacteriaceae	12	1.0	91.9	12	1.0	91.4	12	0.9	92.5
Tenericutes, Other	13	1.0	92.9	13	0.9	92.3	13	0.8	93.3
Firmicutes, Erysipelotrichaceae	14	0.9	93.8	11	1.2	93.5	19	0.3	93.6
Proteobacteria, other *Alphaproteobacteria*	15	0.7	94.5	14	0.9	94.4	18	0.4	94.0
SR1, Other	16	0.7	95.2	15	0.7	95.1	11	1.0	95.0
Tenericutes, Anaeroplasmataceae	17	0.6	95.8	16	0.6	95.7	17	0.4	95.4
Tenericutes, vadinHA31	18	0.5	96.3	18	0.5	96.2	22	0.1	95.5

Firmicutes, *Catabacteriaceae*	19	0.4	96.7	17	0.6	96.8	15	0.6	96.1
TM7, F16	20	0.4	97.1	20	0.4	97.2	23	0.1	96.2
Chloroflexi, Anaerolinaceae	21	0.3	97.4	23	0.0	97.2	20	0.3	96.5
Cyanobacteriaceae, Other	22	0.3	97.7	19	0.4	97.6	16	0.5	97.0
Verrucomicrobia, Other	23	0.3	98.0	22	0.3	97.9	14	0.7	97.7
