# Supplementary material for: Simultaneous Amplicon Sequencing to Explore Co-Occurrence Patterns of Bacterial, Archaeal and Eukaryotic Microorganisms in Rumen Microbial Communities
Source: PLoS One. 2013 Feb 8;8(2):e47879. doi: 10.1371/journal.pone.0047879 (PMC3568148; doi:10.1371/journal.pone.0047879)
Supplement: Table S4 — Comparison of abundances of archaeal clades detected using 3 different primer sets (ArL, ArS, and ArBa) in all analyzed samples. (PDF) [file pone.0047879.s011.pdf]

Table S4. Comparison of abundances of archaeal clades detected using 3 different primer sets (ArL, ArS, and ArBa) in all analyzed samples. Comparison of primer pairs targeting methanogenic archaea in regards to their coverage of major clades. Clades are ranked according to their abundances using primer pair ArL and added up in that order for primer pairs ArS and ArBa to give a cumulative total (Sum). *Mbb.* = *Methanobrevibacter*.

**Clade**	**ArL**			**ArS**			**ArBa**		
	**Rank**	**Abundance**	**Sum**	**Rank**	**Abundance**	**Sum**	**Rank**	**Abundance**	**Sum**
		**[%]**	**[%]**		**[%]**	**[%]**		**[%]**	**[%]**
Mbb. ruminantium	1	37.2	37.2	1	36.0	36.0	2	33.2	33.2
Mbb. gottschalkii	2	34.0	71.2	2	35.2	71.2	1	43.6	76.8
Rumen Cluster C	3	17.6	88.8	4	11.2	82.4	4	6.2	83.0
Methanosphaera	4	9.8	98.6	3	16.9	99.3	3	15.6	98.6
Methanosarcina	5	1.0	99.6	8	0.11	99.4	8	0.07	98.7
Mbb. smithii	6	0.14	99.7	-	-	99.4	-	-	98.7
Sulfolobus	7	0.12	99.8	-	-	99.4	-	-	98.7
Methanobacterium	8	0.12	99.9	5	0.17	99.6	6	0.15	98.8
Methanimicrococcus	9	0.11	100	6	0.16	99.7	7	0.11	98.9
Methanocorpusculum	10	0.03	100	-	-	99.7	-	-	98.9

Methanomicrobium	11	0.02	100	11	0.01	99.8	-	-	98.9
Mbb. wollinii	12	0.01	100	9	0.04	99.8	-	-	98.9
Mbb. arboriphilus	-	-	100	7	0.13	99.9	5	1.1	100
Mbb. woesii	-	-	100	10	0.02	99.9	-	-	100
Methanoculleus	-	-	100	12	0.001	99.9	-	-	100
