# Supplementary material for: Simultaneous Amplicon Sequencing to Explore Co-Occurrence Patterns of Bacterial, Archaeal and Eukaryotic Microorganisms in Rumen Microbial Communities
Source: PLoS One. 2013 Feb 8;8(2):e47879. doi: 10.1371/journal.pone.0047879 (PMC3568148; doi:10.1371/journal.pone.0047879)
Supplement: Text S1 — Run-to-run variation. (PDF) [file pone.0047879.s012.pdf]

Text S1. Run-to-run variation. To evaluate the degree of variation between different 454 Titanium sequencing runs, partial bacterial 16S rRNA genes in 11 rumen samples were amplified once with primer pair BaL and analyzed in two different pyrosequencing runs. DNA was extracted from two subsamples of one rumen sample, so that 12 DNA samples were analyzed in total. The differences in relative abundances of the bacterial groups detected were compared across all 12 DNA samples and the two sequencing runs using the Bray-Curtis dissimilarity metric in QIIME (family level, only taxa with ≥1% abundance included). The between-run variation, measured as the dissimilarity between two identical DNA samples in two different runs (mean dissimilarity 5.5% ± 0.7% (standard deviation)), was smaller than when the same rumen sample was extracted in duplicate and amplicons analyzed in the same 454 run (compare S4SG1PN and S4SG2PN in Figure S5; 6.3% ± 1.2%). Apparently, biases introduced by DNA extraction and amplification of target genes are larger than the bias introduced by the sequencing step, at least for these two runs. Importantly, however, run-to-run variation between two identical DNA samples in different runs was much smaller than the dissimilarity between two unrelated samples in the same run (mean dissimilarity 27.3% ± 9.8%), which is important if data from different runs are to be compared as datasets accumulate.
