# Supplementary material for: Simultaneous Amplicon Sequencing to Explore Co-Occurrence Patterns of Bacterial, Archaeal and Eukaryotic Microorganisms in Rumen Microbial Communities
Source: PLoS One. 2013 Feb 8;8(2):e47879. doi: 10.1371/journal.pone.0047879 (PMC3568148; doi:10.1371/journal.pone.0047879)
Supplement: Text S2 — Diversity and coverage of bacterial and archaeal communities analyzed with different primer combinations. (PDF) [file pone.0047879.s013.pdf]

Text S2. Diversity and coverage of bacterial and archaeal communities analyzed with different primer combinations. Sequencing results obtained from different sets of bacteria-specific primer pairs (Ba9F-Ba515Rmod1 [BaL; V1-V3] and Ba27F-Ba338R [BaS; V1-V2]) and archaea-specific primer pairs (Ar344F-Ar519R [ArS; V3] and Ar915aF-Ar1386R [ArL; V6-V8]) were compared to a primer pair that targets genes of both domains simultaneously (ArBa515F-ArBa806R [ArBa; V4]; [1]). After quality check, a total of 481,492 sequences were obtained from the first and second sequencing run (see Materials and Methods). On average (± SD), we obtained 6,245 ± 2,194 sequences per sample for primer pair BaL, 7,209 ± 1,793 sequences per sample for primer pair ArL, 4,438 ± 487 sequences per sample for primer pair BaS, and 5,474 ± 1,621 sequences per sample for primer pair ArS. For the ArBa primer pair, we simultaneously obtained an average of 15,932 ± 4,341 sequences per sample for the bacteria and 827 ± 487 sequences per sample for the archaea. Comparisons were carried out at a phylogenetic level addressing diversity (number of phylogenetic groups; Table S2), coverage and abundance of OTUs (Tables S3, S4), and the similarities of samples among each other (Figures S2, S3).

Libraries generated using the three primer pairs that amplified 16S rRNA genes from members of the domain Bacteria clustered by sample rather than by primer pair, indicating that all primer pairs resulted in similar bacterial community compositions (Figures S2A, S2B, S3A). The mean between-primer/within-sample dissimilarity (12.3 ± 4.1%) was significantly lower than the mean within-primer/between-sample dissimilarity (27.0 ± 10.1%; $P = 1.5 \times 10^{-29}$). These results suggest that the three primer pairs covering different parts of variable regions V1-V4 of the bacterial 16S rRNA gene produce similar data for rumen bacterial community composition. Similar results were obtained by Wu *et al.*, who used slightly different primer sequences that targeted the V1-V2 and V1-V3 (and other) regions of the 16S rRNA gene [2].

In contrast, when samples were amplified with different primer pairs targeting the domain Archaea, the three libraries generated from each of the samples clustered considerably further apart from each other than did bacterial communities from the same samples (Figures S2C, S2D, S3B). Even so, the mean between-primer/within-sample dissimilarity (13.2 ± 5.3%) was still significantly lower than the within-primer/between-sample dissimilarity (21.7% ± 10.0%; $P = 3.7 \times 10^{-11}$). Pairwise t-tests of the 4 dominant groups of rumen methanogens (*Methanobrevibacter ruminantium* and its relatives, *M. gottschalkii* and its relatives, *Methanosphaera stadtmanae*, and Rumen Cluster C as defined by Janssen and Kirs [3]) revealed that the different primer pairs were significantly biased for or against several of these groups (Figure S6). Thus, comparison of data sets obtained using different primer pairs should be avoided.

REFERENCES

1. Caporaso JG, Lauber CL, Walters WA, Berg-Lyons D, Lozupone CA, et al. (2011) Global patterns of 16S rRNA diversity at a depth of millions of sequences per sample. Proceedings of the National Academy of Sciences of the United States of America 108: 4516-4522.

2. Wu GD, Lewis JD, Hoffmann C, Chen Y-Y, Knight R, et al. (2010) Sampling and pyrosequencing methods for characterizing bacterial communities in the human gut using 16S sequence tags. BMC Microbiology 10: Article No. 206.

3. Janssen PH, Kirs M (2008) Structure of the archaeal community of the rumen. Applied & Environmental Microbiology 74: 3619-3625.
