# Supplementary material for: Simultaneous Amplicon Sequencing to Explore Co-Occurrence Patterns of Bacterial, Archaeal and Eukaryotic Microorganisms in Rumen Microbial Communities
Source: PLoS One. 2013 Feb 8;8(2):e47879. doi: 10.1371/journal.pone.0047879 (PMC3568148; doi:10.1371/journal.pone.0047879)
Supplement: Text S3 — Estimation of the proportion of archaea obtained with the non-specific ArBa primer pair using qPCR and pyrosequencing libraries. (PDF) [file pone.0047879.s014.pdf]

Text S3. Estimation of the proportion of archaea obtained with the non-specific ArBa primer pair using qPCR and pyrosequencing libraries. The ratio of bacteria to archaea obtained in the pyrosequencing library generated with the ArBa primer pair was validated by qPCR of 16S rRNA genes from bacteria and archaea in the same samples (Figure S7). The pyrosequencing libraries showed a significantly higher average abundance of archaea (4.9% ± 2.1%; bacteria: 95.1% ± 2.1%) than qPCR (1.3% ± 0.7%; bacteria: 98.7% ± 0.7%). A similarly high abundance of the archaeal component was also reported by Brulc *et al.* using a primer-independent metagenomics approach [1]. The overall trend of bacterial, and thus archaeal abundances in the 12 pyrosequencing libraries was correlated with the results obtained from qPCR (Pearson correlation coefficient = 0.83, $P < 0.001$; Figure S7), indicating that generalized between-sample comparisons can be validly made from data generated using the ArBa primer pair.

REFERENCES

1. Brulc JM, Antonopoulos DA, Miller MEB, Wilson MK, Yannarell AC, et al. (2009) Gene-centric metagenomics of the fiber-adherent bovine rumen microbiome reveals forage specific glycoside hydrolases. Proceedings of the National Academy of Sciences of the United States of America 106: 1948-1953.
